# Genetic Associations with Gestational Length and Spontaneous Preterm Birth

**DOI:** 10.1056/NEJMoa1612665

**Published:** 2017-09-06

**Authors:** Ge Zhang, Bjarke Feenstra, Jonas Bacelis, Xueping Liu, Lisa M. Muglia, Julius Juodakis, Daniel E. Miller, Nadia Litterman, Pan-Pan Jiang, Laura Russell, David A. Hinds, Youna Hu, Matthew T. Weirauch, Xiaoting Chen, Arun R. Chavan, Günter P. Wagner, Mihaela Pavličev, Mauris C. Nnamani, Jamie Maziarz, Minna K. Karjalainen, Mika Rämet, Verena Sengpiel, D Frank Geller, Heather A. Boyd, Aarno Palotie, Allison Momany, Bruce Bedell, Kelli K. Ryckman, Johanna M. Huusko, Carmy R. Forney, Leah C. Kottyan, Mikko Hallman, Kari Teramo, Ellen A. Nohr, George Davey-Smith, Mads Melbye, Bo Jacobsson, Louis J. Muglia

**Affiliations:** Division of Human Genetics, Cincinnati Children’s Hospital Medical Center, Cincinnati, OH, USA; Center for Prevention of Preterm Birth, Perinatal Institute, Cincinnati Children’s Hospital Medical Center and March of Dimes Prematurity Research Center Ohio Collaborative, Cincinnati, OH, USA; Department of Epidemiology Research, Statens Serum Institut, Copenhagen, Denmark; Department of Obstetrics and Gynecology, Sahlgrenska University Hospital Östra (East), Gothenburg, Sweden; Department of Epidemiology Research, Statens Serum Institut, Copenhagen, Denmark; Center for Prevention of Preterm Birth, Perinatal Institute, Cincinnati Children’s Hospital Medical Center and March of Dimes Prematurity Research Center Ohio Collaborative, Cincinnati, OH, USA; Department of Obstetrics and Gynecology, Institute of Clinical Sciences, Sahlgrenska Academy, University of Gothenburg, Gothenburg, Sweden; Center for Autoimmune Genomics and Etiology, Cincinnati Children's Hospital Medical Center, Cincinnati, OH, USA; 23andMe, Inc. Mountain View, CA, USA; 23andMe, Inc. Mountain View, CA, USA; 23andMe, Inc. Mountain View, CA, USA; 23andMe, Inc. Mountain View, CA, USA; 23andMe, Inc. Mountain View, CA, USA; Center for Autoimmune Genomics and Etiology; Divisions of Biomedical Informatics and Developmental Biology, Cincinnati Children's Hospital Medical Center, Cincinnati, OH, USA; Center for Autoimmune Genomics and Etiology, Cincinnati Children's Hospital Medical Center, Cincinnati, OH, USA; Department of Ecology and Evolutionary Biology, Yale University, New Haven, CT, Yale Systems Biology Institute, West Haven, CT, USA; Department of Ecology and Evolutionary Biology, Yale University, New Haven, CT, Yale Systems Biology Institute, West Haven, CT, Department of Obstetrics, Gynecology and Reproductive Sciences, Yale Medical School, New Haven, CT, Department of Obstetrics and Gynecology, Wayne State University, Detroit, MI, USA; Center for Prevention of Preterm Birth, Perinatal Institute, Cincinnati Children’s Hospital Medical Center and March of Dimes Prematurity Research Center Ohio Collaborative, Cincinnati, OH, USA; Department of Ecology and Evolutionary Biology, Yale University, New Haven, CT, Yale Systems Biology Institute, West Haven, CT, USA; Department of Ecology and Evolutionary Biology, Yale University, New Haven, CT, Yale Systems Biology Institute, West Haven, CT, USA; PEDEGO Research Unit and Medical Research Center Oulu, University of Oulu and Department of Children and Adolescents, Oulu University Hospital, Oulu, Finland; PEDEGO Research Unit and Medical Research Center Oulu, University of Oulu and Department of Children and Adolescents, Oulu University Hospital, Oulu, Finland; Department of Obstetrics and Gynecology, Sahlgrenska University Hospital Östra (East), Gothenburg, Sweden; Department of Epidemiology Research, Statens Serum Institut, Copenhagen, Denmark; Department of Epidemiology Research, Statens Serum Institut, Copenhagen, Denmark; Analytic and Translational Genetics Unit, Department of Medicine, Massachusetts General Hospital, Boston, MA, USA.; Program in Medical and Population Genetics, The Broad Institute of MIT and Harvard, Cambridge, MA, USA; The Stanley Center for Psychiatric Research, The Broad Institute of MIT and Harvard, Cambridge, MA, USA. Institute for Molecular Medicine Finland, University of Helsinki, Helsinki, Finland. ; Psychiatric & Neurodevelopmental Genetics Unit, Department of Psychiatry, Massachusetts General Hospital, Boston, MA, USA.; Department of Neurology, Massachusetts General Hospital, Boston, MA, USA; Department of Pediatrics, Carver College of Medicine, University of Iowa, Iowa City, IA, USA; Department of Pediatrics, Carver College of Medicine University of Iowa, Iowa City, IA, USA; Department of Epidemiology, College of Public Health and Department of Pediatrics, Carver College of Medicine, University of Iowa, Iowa City, IA, USA; PEDEGO Research Unit and Medical Research Center Oulu, University of Oulu and Department of Children and Adolescents, Oulu University Hospital, Oulu, Finland; Center for Prevention of Preterm Birth, Perinatal Institute, Cincinnati Children’s Hospital Medical Center and March of Dimes Prematurity Research Center Ohio Collaborative, Cincinnati, OH, USAaww; Center for Autoimmune Genomics and Etiology, Cincinnati Children's Hospital Medical Center, Cincinnati, OH, USA; Center for Autoimmune Genomics and Etiology, Cincinnati Children's Hospital Medical Center, Cincinnati, OH, USA; PEDEGO Research Unit and Medical Research Center Oulu, University of Oulu and Department of Children and Adolescents, Oulu University Hospital, Oulu, Finland; Obstetrics and Gynecology, University of Helsinki and Helsinki University Hospital, Helsinki, Finland; Research Unit of Gynaecology & Obstetrics, Institute of Clinical Research, University of Southern Denmark, Odense, Denmark; Medical Research Council Integrative Epidemiology Unit (IEU) at the University of Bristol, School of Social and Community Medicine, University of Bristol, Oakfield House, Oakfield Grove, Bristol, United Kingdom; Department of Epidemiology Research, Statens Serum Institut, Copenhagen, Denmark; Department of Clinical Medicine, University of Copenhagen, Copenhagen, Denmark; Department of Medicine, Stanford University School of Medicine, Stanford, CA, USA; Department of Obstetrics and Gynecology, Sahlgrenska Academy, University of Gothenburg, Gothenburg, Sweden; Department of Genetics and Bioinformatics, Area of Health Data and Digitalisation, Norwegian Institute of Public Health, Oslo, Norway; Division of Human Genetics, Center for Prevention of Preterm Birth, Perinatal Institute, Cincinnati Children’s Hospital Medical Center and March of Dimes Prematurity Research Center Ohio Collaborative, Cincinnati, OH, USA

**Keywords:** Gestational length, preterm birth, genomewide association, single nucleotide polymorphism

## Abstract

**Background:**

Despite evidence that genetic factors contribute to gestational length and preterm birth, robust associations with genetic variants have not been identified. We hypothesized that analyzing larger data sets with gestational length information by genomewide association would reveal trait-influencing variants.

**Methods:**

We performed a genomewide association study in a discovery data set of 43,568 women of European ancestry from 23andMe, Inc., for gestational length as a continuous trait and for term or preterm (<37 weeks) birth as a dichotomous outcome. We used three Nordic data sets (8,643 women) for replication of 14 genomic loci achieving either genomewide (*P* < 5×10^-8^) or suggestive association (*P* < 1×10^-6^).

**Results:**

In the discovery stage, for gestational length, four loci (*EBF1*, *EEFSEC*, *AGTR2* and *WNT4*) achieved genomewide significance, all of which were replicated in the Nordic data sets. Functional analysis of the *WNT4* locus indicated the likely causative variant alters the binding of ESR1. *ADCY5* and *RAP2C*, which had suggestive significance in the discovery stage, were significantly replicated and achieved genomewide significance in joint analysis. Common variants in *EBF1*, *EEFSEC* and *AGTR2* were also associated with preterm birth with genomewide significance. Analysis of mother-infant dyads indicated that these findings likely resulted from maternal genome actions.

**Conclusions:**

Our study is the first to identify maternal genetic variants robustly associated with gestational length and preterm birth. Roles of these loci in uterine development, maternal nutrition, and vascular control support their mechanistic involvement and create opportunities to investigate new risk factors for prevention of preterm birth.

## Introduction

Preterm birth (defined as birth before 37 completed weeks of gestation) affects 9.6% of pregnancies in the United States^1^ and over 15 million pregnancies worldwide each year. It is the leading global cause of mortality in children under five years of age.^2,3^ The majority of preterm births arise by the spontaneous, idiopathic onset of uterine contractions or rupture of fetal membranes.^4^ Despite the considerable morbidity and mortality arising from preterm birth, few interventions have proven effective in limiting its occurrence. The limited progress in preterm birth prevention may arise from the lack of understanding of the pathways regulating the timing of birth including the normal length of gestation.^5,6^ A substantial body of evidence has accumulated demonstrating a contribution of genetic factors in gestational length and preterm birth risk.^7^ For example, twin and family studies suggest that 30-40% of the variation in birth timing, or risk for preterm birth, arises from genetic factors, largely but not exclusively residing in the maternal genome.^8-12^

Preterm birth, and gestational length in general, is a complicated phenotype with contributions from two genomes – maternal and fetal – that may have separate or interacting contributions. Furthermore, different genotypes may predispose to preterm birth at different gestational ages. Finally, defining preterm birth as a dichotomous trait based upon a somewhat arbitrary cutoff of 37 weeks, rather than time of birth for a specified level of fetal maturity or as a continuous trait, limits data interpretation. Therefore, defining the genetic variants associated with gestational length (a quantitative trait) as well as preterm birth (a dichotomous trait), will both yield important new insights. Further, analyzing gestational length as a continuous trait increases the power to detect associations that is limited when traits are dichotomized.^13^ Control of timing of birth is multifactorial, and common polymorphisms involved in gestational length or preterm birth risk are likely to individually be of small effect size. Nonetheless, the insights they provide into essential genes and pathways may open novel avenues for intervention.^14^ However, for genomewide association studies to reveal robustly associated variants, large sample sizes are required,^15^ and particularly so for preterm birth given the complexity of the phenotype. To date, individual genomewide association studies of spontaneous preterm birth have included on the order of 1,000 case mothers or infants with control groups of similar size, but no replicated genomewide significant loci have yet emerged.^16-18^

To overcome previous sample size limitations, we leverage data on gestational length and preterm birth in a large sample of women of European ancestry (approximately 44,000) collected as part of genotyping and phenotyping efforts by 23andMe, Inc., a genetics company. We then selected the top loci (*P* < 1×10^-6^) and performed replication analyses for gestational length and preterm birth in three data sets of Nordic women (8,643). Further, we provide evidence indicating the observed effect was due to an action in the maternal genome and provide functional data implicating the causative SNP underlying the *WNT4* locus.

## Methods

We performed a two-stage genomewide association study to discover and replicate genetic loci associated with gestational length and preterm birth. In the discovery stage, we performed genomewide association analyses on 43,568 European-ancestry females identified among 23andMe’s research participants. In the replication stage, the top significant loci from the discovery stage analyses were tested in three birth data sets collected from Nordic countries (Finland, Denmark, and Norway).

### Discovery stage

Women in the discovery data set were participants in 23andMe’s research program. All women provided informed consent and answered surveys online following a human subjects protocol, reviewed and approved by Ethical & Independent Review Services, a private institutional review board (http://www.eandireview.com). Unrelated women of European ancestry who self-reported gestational length of their first live singleton birth were included in the analysis. Categories of preterm birth addressed on the survey were 1) spontaneous preterm labor, 2) planned or required delivery for medical reasons, 3) cervical problems, 4) other, or 5) none of the above. Women with a medical indication for their preterm delivery were excluded from the study; those that did not specify a medical indication on the survey were retained to optimize sample size. Preterm birth status was determined based on dichotomization of self-reported gestational length (preterm < 37 weeks; term ≥37 weeks). For those in the preterm group, 96.8% of women responded to the question regarding mode of delivery. For the term birth group, we ascertained information on aggregate outcomes of all pregnancies, and could not unambiguously determine spontaneous or medically indicated birth at more than 37 weeks.

DNA extraction and genotyping were performed on saliva samples by the National Genetics Institute. To minimize the effects of population stratification, we restricted analyses to women with >97% European ancestry, as determined through an analysis of local ancestry.^19^ Participant genotype data were imputed against the 1000 Genomes Phase1 reference haplotypes.^20^

Single-marker genetic associations with gestational length and preterm birth were tested by linear regression or logistic regression, respectively, using imputed allelic dosage data assuming additive allelic effects. Maternal age and the top five principal components to account for residual population structure were included as covariates.

We clustered SNPs into association regions (or loci). Specifically, we defined association regions by first identifying SNPs with *P* < 1×10^-4^, then grouping these into a region if they were adjacent to each other (<250kb). The SNP with smallest *P* value within each region was chosen as the index SNP. Regions that achieved suggestive significance (*P* < 1×10^-6^) were tested in the replication stage.

### Replication stage

We used the data of 8,643 mothers from three independent Nordic birth studies (Table S1) of singleton pregnancies with spontaneous onset of labor. Briefly, the Finnish study (FIN) consisted of nearly 900 mothers and their infants recruited from the Helsinki University Hospital between 2004 and 2014 with gestational length confirmation by early ultrasound at 10-13 weeks of gestation. The Mother Child Cohort of Norway (MoBa) is a nationwide pregnancy cohort study administered by the Norwegian Institute of Public Health.^21^ The genotype data were derived from a genomewide association study of preterm birth, with gestational length determination by second trimester ultrasound in more than 95% of participants.^22^ For the current study, 1,834 mothers and 1,143 infants that passed QC were included in the analysis. The Danish National Birth Cohort (DNBC) data is a cohort including mothers and their children from more than 100,000 pregnancies recruited between 1996 and 2002.^23^ Gestational length in this cohort was assigned by combining all available information from multiple sources: self-reported date of last menstrual period, self-reported delivery date, and gestational length at birth registered in the Medical Birth Register and the National Patient Register. The genotype data were derived from two genomewide association studies of preterm birth^24^ and obesity,^25,26^ respectively. In the current study, data from 5,921 mothers and 2,130 infants that passed QC were analyzed. 

Genotyping of the Nordic studies was conducted using various SNP arrays as previously described.^27^ Similar genotype QC procedures were used across the three studies. Subjects of non-European-ancestry were identified and excluded using principal components analysis (PCA). Genomewide imputation for the replication data sets was conducted using the reference haplotypes extracted from the Phase I 1000 Genomes Project.^20^

Single-marker genetic association tests were conducted in each replication data set, using regression methods and imputation dosage similar to the discovery stage. Genotypic association tests (d.f. = 2) were also performed to examine possible dominance effect. The replication *P* values (inflation adjusted) combining results from the three Nordic data sets were calculated using the fixed-effects inverse-variance method. Significant replication *P* values and the same direction of effect at the index or other significant SNPs (*P* < 1×10^-6^, discovery stage) in the region or their close proxies (*r* ^2^ > 0.8) were regarded as statistical evidence of replication of a putative locus. The significance level of each region was corrected by the effective number of independent SNPs tested in the region and the total number of regions that underwent replication attempts (Table S5 and Supplementary Text). A region was considered successfully replicated and genomewide significant after replication if the most significant replication *P* value was below the significance level and had a combined discovery and replication *P* value less than 5×10^-8^. 

We also performed association tests in 4,090 infant samples and joint maternal/fetal genetic association analysis in 3,184 (FIN: 769; MoBa: 1019 and DNBC: 1396) mother/infant pairs from the Nordic data sets to evaluate whether the observed significant associations were likely to be of maternal or fetal origin.

### Functional annotation and other statistical analyses

We checked whether the SNPs associated with gestational length or preterm overlap with previously reported genomewide association SNPs in the GWAS catalog^28^ and used the GTEx^29^ database to search for associations with tissue-specific gene expression. We examined whether multiple independent variants at a given locus influenced birth timing by an approximate conditional and joint multiple-SNP (COJO) analysis.^30^ We estimated the fraction of phenotype variance in the replication data sets explained by all common SNPs^31^ by GCTA^32^ or sets of SNPs associated at different significance thresholds in the discovery cohort using a genetic score approach.^33^ We also performed gene-centric associations and gene-set enrichment analyses. Detailed description of these analyses and associated results are described in the Supplementary Text. 

### Functional follow-up

We performed experimental functional follow-up of the *WNT4* locus, one of the most significant loci with plausible functional relevance in pregnancy. First we examined the expression level of *WNT4* in human endometrial stromal cells, before and after decidualization using mRNA-seq technology. We predicted specific transcription factors binding using a Bioinformatic approach and studied the presence of H3K4me3 marks and open chromatin domains overlapping the hypothetical causal SNP by ChIP-seq and ATAC-seq, respectively. We performed electrophoretic mobility shift assays (EMSA) to determine whether the variant differentially affected specific transcription factor binding. Detailed description of these analyses can be found in the Supplementary Text (Functional analyses of the *WNT4* locus).

## Results

### Study data sets

The discovery data set included 43,568 women identified through 23andMe (Table S1). Most of the women (86.8%, N=37,803) delivered at term (37 to 42 weeks); 7.6% (N=3,331) delivered preterm (<37 weeks) and 5.6% (N=2,434) delivered post-term (>42 weeks) (Figure S1 and Table S2). Maternal age was strongly associated with gestational length (*P* = 2.3×10^-41^) with older mothers having shorter gestational length (Table S3).

Three Nordic birth studies^27^ were used in combination for replication. In total, phenotype and genotype data were available from 8,643 mothers and 4,090 infants (Table S1). These data sets were case/control studies, in which samples from preterm births were enriched and samples with post-term or close to the preterm-term boundary (37-38 weeks) were excluded (Figure S2). In these studies infant gender and maternal height were associated with gestational length (Table S4). 

### Discovery stage findings in mothers

**Figure 1. Manhattan plots of discovery stage genomewide-associated results.**
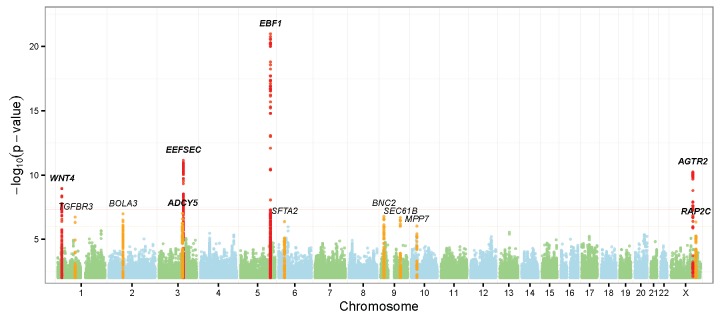

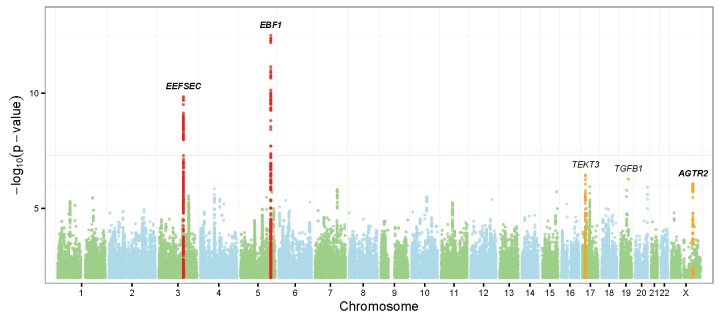
Top: gestational length as quantitative trait; bottom: preterm birth as dichotomous trait. Regions reached genome wide significance (*P* < 5×10^-8^) and suggestive significance (*P* < 1×10^-6^) were highlighted in red and orange respectively. The six replicated loci were highlighted in bold.

Single-marker association tests were performed across 15,635,593 SNPs that passed the 23andMe QC (Supplementary Methods). We focused our analysis on 9,042,878 markers with MAF>0.01. Test results were adjusted for genomic inflation factors (Figure S3). For gestational length, 12 loci were identified with *P* < 1×10^-6^ (suggestive significance). Of these, four had an association P < 5×10^-8^ (Figure 1A, Table 1 and Table S5). For preterm birth, 5 loci were identified with P < 1×10-6, two of which achieved genomewide significance (Figure 1B, Table 1 and Table S5). The top three loci associated with gestational length (*EBF1*, *EEFSEC* and *AGTR2*) shared association loci for preterm birth risk. Altogether, 14 independent loci were taken forward for replication. To confirm the robustness of the association signals, we conducted similar association tests in a subset of discovery subjects who explicitly checked “spontaneous delivery” in the questionnaire (excluding those who did not specify a choice of spontaneous or medically indicated delivery) and the results were similar to those obtained from the full discovery data sets (Table S6).

### Replication of suggestive genomewide-associated loci

For each of the 14 loci from the discovery stage, we examined the replication association signals (*P* value and direction of effect) at the index SNP and other SNPs with *P* < 1×10^-6^ in the discovery stage, and their close proxies (*r* ^2^ > 0.8). Six loci (Table 1 and Table S7, S8) replicated given a significance threshold adjusted for the effective number of independent SNPs at a locus as well as the number of loci tested (Table S5) and the direction of the effect. The 6 loci include *EBF1*, *EEFSEC*, and *AGTR2*, which were associated with both gestational length and preterm birth; *WNT4*, *ADCY5* and *RAP2C*, which were associated with gestational length but not with preterm birth at the significance level for genomewide discovery (*P* < 1×10^-6^). In addition, associations of the *BOLA3* locus with gestational length, and the *TEKT3* and the *TGFB1* loci with preterm birth showed marginal significance (*P* < 0.05). At the *EBF1*, *EEFSEC*, *AGTR2*, *WNT4* and *RAP2C* loci, the most significant SNPs in the replication stage were either same as or in substantial LD (*r* ^2^ > 0.6) with the most significant SNPs in discovery stage. However, at the *ADCY5* locus, the LD between the most significant SNPs in replication stage and the discovery stage is less substantial (*r* ^2^ < 0.4). SNPs at the *EEFSEC* locus showed nominally significant dominant effects (*P*.dom < 0.05) (Table S7, S8). 

**Table 1. Discovery and replication of loci associated with gestational length or preterm birth.** For each locus, the most significant SNP in discovery stage (index SNP) and the most significant SNP in replication stage are shown.

No	Char	Genes@	SNP Information#			Discovery Phase					Replication Phase				Joint analysis
			rs	pos	A/B	Freq	Eff$	*P*-value%	Rank&	*r* ^2^	Freq	Eff$	*P*-value%	Directions*	*P*–value%
***Gestational age***															
**1**	**5**	***EBF1***	**rs2963463**	**157895049**	**C/T**	**0.272**	**-1.29**	**1.0E-21**	**1**	****	**0.264**	**-1.11**	**0.0017**	**+-+**	**7.7E-24**
			***rs2946171***	***157921940***	***T/G***	***0.219***	***-1.24***	***1.1E-17***	***24***	***0.71***	***0.206***	***-1.46***	***0.00014***	***+-+***	***8.1E-21***
**2**	**3**	***EEFSEC***	**rs2955117**	**127881613**	**G/A**	**0.286**	**0.911**	**7.2E-12**	**1**	****	**0.279**	**1.33**	**0.00016**	**+++**	**9.5E-15**
			***rs200745338***	***127869457***	***D/I***	***0.237***	***0.986***	***1.5E-11***	***8***	***0.72***	***0.232***	***1.91***	***7.6E-07***	***+++***	***7.5E-16***
**3**	**X**	***AGTR2***	**rs201226733**	**115164770**	**I/D**	**0.422**	**-0.820**	**5.7E-11**	**1**	****	**0.420**	**-1.67**	**9.2E-08**	**+++**	**7.2E-16**
			***rs5950491***	***115129714***	***C/A***	***0.423***	***-0.826***	***6.8E-11***	***5***	***0.92***	***0.425***	***-1.75***	***4.7E-08***	***+++***	***6.6E-16***
**4**	**1**	***WNT4***	**rs56318008**	**22470407**	**C/T**	**0.139**	**1.05**	**1.2E-09**	**1**	****	**0.153**	**2.27**	**1.8E-07**	**+++**	**3.4E-14**
			***rs12037376***	***22462111***	***G/A***	***0.145***	***1.00***	***4.5E-09***	***4***	***0.91***	***0.157***	***2.41***	***2.1E-08***	***+++***	***5.6E-14***
**5**	**3**	***ADCY5***	**rs4383453**	**123068359**	**G/A**	**0.200**	**-0.808**	**9.6E-08**	**1**	****	**0.197**	**-0.587**	**0.15**	**-++**	**3.7E-08**
			***rs9861425***	***123072883***	***A/C***	***0.453***	***-0.598***	***6.1E-07***	***5***	***0.34***	***0.470***	***-1.38***	***9.5E-06***	***+++***	***4.2E-10***
6	2	*BOLA3*	rs4853012	74361290	G/A	0.141	-0.920	1.1E-07	1		0.145	-0.355	0.42	-++	1.6E-07
			*rs17009553*	*74220035*	*G/A*	*0.0567*	*-1.28*	*1.1E-06*	*6*	*0.13*	*0.0565*	*-2.11*	*0.0020*	*-++*	*1.6E-08*
7	9	*BNC2*	rs717267	16408826	G/A	0.399	-0.637	1.7E-07	1		0.423	-0.12	0.70	+-+	5.3E-07
			*rs9298764*	*16431230*	*A/G*	*0.456*	*-0.55*	*5.2E-06*	*19*	*0.81*	*0.474*	*-0.443*	*0.16*	*+-+*	*2.0E-06*
8	1	*TGFBR3*	rs4658267	92240753	C/A	0.319	0.679	1.9E-07	1		0.319	0.0562	0.87	-++	8.7E-07
			*rs4658265*	*92240685*	*C/T*	*0.312*	*0.662*	*4.8E-07*	*2*	*0.91*	*0.311*	*0.125*	*0.71*	*-++*	*1.4E-06*
9	9	*SEC61B*	rs182704	*102068912*	*T/C*	*0.344*	*0.658*	*2.1E-07*	*1*		*0.397*	*0.145*	*0.67*	*--+*	*5.4E-07*
10	6	*SFTA2*	rs2532929	30897774	A/G	0.397	-0.622	4.2E-07	1		0.402	-0.0486	0.88	+-+	1.8E-06
			*rs2532926*	*30898441*	*A/G*	*0.363*	*-0.556*	*9.0E-06*	*3*	*0.87*	*0.374*	*-0.0653*	*0.84*	*+-+*	*2.5E-05*
**11**	**X**	***RAP2C***	**rs200879388**	***131300571***	***I/D***	***0.351***	***-0.662***	***4.5E-07***	***1***		***0.364***	***-1.1***	***0.00092***	***+++***	***3.4E-09***
12	10	*MPP7*	rs2253165	28337017	A/G	0.440	-0.594	9.3E-07	1		0.429	0.454	0.15	+--	4.5E-05
			*rs2245244*	*28316456*	*T/C*	*0.438*	*-0.561*	*3.7E-06*	*2*	*0.86*	*0.428*	*0.507*	*0.11*	*+--*	*0.00019*
***Preterm birth***															
**1**	**5**	***EBF1***	**rs2963463**	**157895049**	**C/T**	**0.272**	**1.23**	**3.2E-13**	**1**	****	**0.265**	**1.13**	**0.0015**	**+-+**	**4.5E-15**
			***rs2946169***	***157918959***	***C/T***	***0.217***	***1.22***	***1.1E-10***	***19***	***0.68***	***0.207***	***1.16***	***0.00055***	***+-+***	***2.2E-13***
2	3	***EEFSEC***	**rs201450565**	**128058610**	**D/I**	**0.233**	**0.810**	**1.4E-10**	**1**		**0.135**	**0.824**	**0.0017**	**+++**	**1.9E-12**
			***rs200745338***	***127869457***	***D/I***	***0.237***	***0.829***	***9.0E-09***	***95***	*****0.24 *	***0.232***	***0.797***	***3.5E-07***	***+++***	***3.3E-14***
3	17	*TEKT3*	rs7217780	15191024	T/C	0.336	1.15	3.5E-07	1		0.341	1.09	0.025	+++	4.9E-08
			*rs179521*	*15173221*	*C/A*	*0.359*	*1.13*	*3.8E-06*	*11*	*0.81*	*0.357*	*1.10*	*0.012*	*+++*	*1.6E-07*
4	19	*TGFB1*	rs11466328	41851042	*G/A*	*0.0288*	*0.567*	*5.3E-07*	*1*		*0.0311*	*0.711*	*0.038*	*+++*	*5.5E-07*
**5**	**X**	***AGTR2***	**rs201386833**	**115164281**	**D/I**	**0.410**	**1.15**	**8.5E-07**	**1**		**0.41**	**1.18**	**2.3E-06**	**+++**	**1.0E-11**
			***rs5950506***	***115175748***	***G/A***	***0.420***	***1.14***	***1.2E-06***	***10***	***0.92***	***0.418***	***1.18***	***1.E-06***	***+++***	***1.1E-11***

For each suggestive locus (*P* < 1×10^-6^, discovery stage), the SNP showing the strongest association in the replication stage is shown below the index SNP (the most significant SNP in discovery stage). Only SNPs with *P* < 1×10^-6^ (discovery stage) and their close proxies (*r* ^2^ > 0.8) were tested for replication. Replicated regions are highlighted in bold.

@ For each region, the gene closest to the index SNP was shown.

# SNP positions were based on GRCh37/hg19. Alleles were given based on positive strand of reference genome. Allele B is used as the reference allele for frequency and effect.

$ For gestational length, effect is unstandardized regression coefficient, which shows the estimated changes in gestational days per allele (B). For preterm birth, effect is the estimated odds ratio of the reference allele (B).

% Discovery stage *P*-values were adjusted by inflation factors. The replication stage *P*-values were calculated from the inflation adjusted effect sizes and standard error of the three Nordic studies using fixed-effect meta-analysis. Joint-analysis *P*-values were calculated from 23andMe and combined Nodic studies using the inverse variance method.

* Directions represent whether the effects observed in the three Nordic studies (FIN/MoBa/DNBC) are same (+) or different (-) from the effects estimated from the 23andMe discovery cohort.

& For each locus, the rank (based on the *P*-value in discovery stage) of the most significant SNP in replication stage (show in italic) together with the *r* ^2^ with the index SNP was provided. The *r* ^2^ was estimated from haplotype data of the Phase 1 1000 Genomes EUR samples.

### Annotation of SNPs at significant loci

A number of SNPs with potentially functional impact (i.e. nonsense, missense and splicing SNPs) are encompassed by the loci we identified as potentially important (Table S5). However, none of those potentially functionally important variants are in close LD (*r* ^2^ >0.8) with SNPs significantly associated with gestational length or preterm birth in the discovery stage. Within these loci, there are SNPs reportedly associated with complex traits (GWAS Catalog^28^) (Table S5). Among these, three previously identified SNPs (rs10934853, rs2999052 and rs2687729) in the *EEFSEC* locus were significantly associated with gestational length and preterm birth. The alleles that were associated with longer gestational length (or reduced risk of preterm birth) have also been associated with increased risk of prostate cancer (rs10934853-A)^34^, reduced risk of hypospadias (rs2999052-C)^35^ and later age of menarche (rs2687729-G).^36,37^ Five significant SNPs in the *WNT4* locus were previously associated with endometriosis,^38^ ovarian cancer^39^ and bone mineral density.^40^ The alleles that increased gestational length in our analysis have also been identified as high-risk alleles for endometriosis, ovarian cancer or low bone mineral density (Table S9). Our eQTL analyses showed that some significant SNPs at the associated loci can significantly influence expression level of nearby genes (*cis*-expression QTLs) based on GTEx data^29^ (Table S10 and S11). 

SNPs at the *ADCY5* locus have been reported to be associated with birth weight^41^ and blood glucose traits.^42^ More recently, a large meta-analysis has revealed SNPs at the *ADCY5*, *WNT4* and *EBF1* loci that are associated with birth weight.^43^ The SNPs at the *ADCY5* and *WNT4* loci appear to influence birth weight through the fetal genome and none of them were in close LD with the SNPs showing significant association with gestational length; while the SNP (rs7729301) at the *EBF1* locus seems to influence birth weight through the maternal effect, and the allele (G) associated with reduced birth weight was also associated with shorter gestational length (Table S5). 

### Maternal or fetal genetic effect

Association analyses of the top regions in the infant samples from our Nordic data sets (Table S12 and S13) yielded weaker associations. The results showed the same direction of effect but smaller effect sizes for the top significantly replicated SNPs (Table S14), supporting the inference that the loci identified in this study are “maternal” loci. The effect sizes estimated from infant samples were highly correlated (ρ = 0.95) and approximately half of the effect sizes estimated from maternal samples (Figure S4), supporting that the effect observed in infants is due to sharing of one maternal allele by descent. In addition, joint association analysis in mother/infant pairs with both maternal and fetal genotypes as predictors demonstrated significant associations exclusively with maternal genotypes but not with fetal genotypes (Table S15), which again indicated the maternal origin of the observed genetic associations.

We also evaluated our findings for detection of allelic heterogeneity, dominance effects, percentage of the variance explained, and gene set enrichment/pathway analyses. These results are presented in the Supplementary Text and include Figures S5-9 and Tables S16-19.

### Functional evidence implicating the *WNT4* locus

The genetic loci we identified fall in noncoding regions of the genome, suggesting that they will affect gene regulation rather than protein function. To dissect the consequences of these variants, knowledge of cell-type context in which they are active is essential. The *WNT4* locus provides an especially attractive region, as unlike the other loci we identified, it implicates a particular tissue context related to its role in pregnancy, the endometrium.^44^ The variants we identified also associate with risk for endometriosis,^45^ and *WNT4* function is critical for decidualization of the endometrium and subsequently implantation and establishment of pregnancy.^46^ Therefore, we sought to analyze the expression of the *WNT4* gene in human endometrial stromal cells, before and after decidualization (Supplementary Methods). Using RNA sequencing, we confirmed a substantial induction of *WNT4* mRNA with decidualization – average of 0.0 transcripts per million (TPM) prior to decidualization in vitro to 29.5 TPM after decidualization in samples run in duplicate from two different endometrial stromal cell lines. 

**Figure 2. ESR1 binding at the WNT4 locus.**
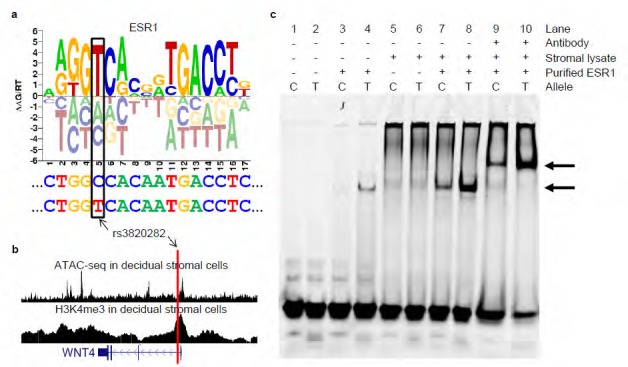
a. The rs3820282 T allele creates a stronger ESR1 binding site. The ESR1 binding motif ‘sequence logo’ (taken from the CisBP web server) illustrates the DNA binding preferences of ESR1. Tall nucleotides above the X-axis indicate DNA bases preferred by ESR1. Bases below the X-axis are disfavored. The sequence located in the *WNT4* promoter is shown below, with the T allele for rs3820282 shown at the bottom. Note that the T allele changes the sequence from C (most disfavored) to T (most preferred). b. rs3820282 overlaps ATAC-seq and H3K4me3 signals in decidual stromal cells at the *WNT4* locus. The red vertical line indicates the position of rs3820282. The location of the *WNT4* gene is depicted at the bottom. Tall blocks indicate exons, medium height blocks indicate UTRs, and thin lines indicate introns. Arrows within introns indicate the direction of transcription. c. Experimental validation of allele-dependent binding of ESR1 to rs3820282 by electrophoretic mobility shift assay (EMSA). Fluorescently-labeled rs3820282 probe with either the C or T allele was incubated with nuclear extracts of decidual stromal endometrial cells in the presence or absence of purified ESR1 and/or antibody against ESR1. Lane pairs indicate C and T alleles. Preferential binding of ESR1 to the T allele is observed through an increased signal intensity of the shifted band. Upper and lower arrows indicate the locations of supershifted and shifted bands, respectively. Left to right - Lanes 1+2: negative control lanes containing only oligos; Lanes 3+4: increased binding of purified ESR1 to the T allele. Lanes 5+6: limited binding in the presence of nuclear extract only, due to low expression of ESR1 in these cells; Lanes 7+8: substantial allelic binding is detected with the addition of purified ESR1 to the nuclear extract; Lanes 9+10: Supershift using an ESR1 antibody

We next sought to identify particular regulatory mechanisms controlling the expression of *WNT4* that might be altered by variants associated with gestational length. To this end, we used the CisBP web server^47^ to predict the specific transcription factors whose binding might be altered by any of the six gestational length-associated variants that localize to the *WNT4* locus. These analyses indicate that rs3820282 (*r* ^2^=0.94 with the index SNP rs56318008), which is located in the first intron of *WNT4* is capable of altering the binding of the estrogen receptor (ESR1). Specifically, the underlying quantitative data from protein binding microarray (PBM) assays^48^ indicate that the minor allele (T) of rs3820282 “creates” a near-perfect half-site for ESR1 (Figure 2) – the PBM-derived E-score for the major allele (C) is 0.09 (no binding), whereas the minor allele (T) is 0.46 (strong binding). Importantly, ESR1 and ESR2 are the only two human nuclear receptors that bind GGTCA half-sites with an "IR3" (Inverted Repeat 3) pattern,^49^ eliminating the ~50 other nuclear receptors from consideration. Further, ChIP-seq peaks for ESR1 are present in four different experiments performed in MCF7 cells^50^, indicating that ESR1 is capable of binding to this locus in a cellular context. We confirmed the presence of H3K4me3 marks and an open chromatin domain by ATAC-seq overlapping rs3820282 (Figure 2b) in an immortalized endometrial stromal cell line (Supplementary Methods), demonstrating that the chromatin over this locus is likely accessible and active in these cells. Importantly, we observed enhanced binding of ESR1 to the T allele of rs3820282 in electrophoretic mobility shift assays, as predicted by the in silico analysis (Figure 2c). Collectively, these data suggest that the likely mechanism underlying the gestational length association in the *WNT4* locus is modulation (via rs3820282) of the binding of ESR1. rs3820282 is also strongly associated with epithelial ovarian cancer^39^ (Table S9), suggesting that this same mechanism might be acting in multiple diseases. 

## Discussion

Our genomewide association study is the first to identify human genetic polymorphisms that are significantly and reproducibly associated with gestational length and preterm birth, the single greatest contributor to mortality in children younger than five years and a common source of morbidity throughout life for those who survive. Our approach demonstrates the utility of using data collected as part of direct-to-consumer genotyping and phenotyping to rapidly assemble large data sets capable of revealing contributing loci in particularly complex phenotypes such as preterm birth where both maternal and fetal genomes, and many genes, are likely to contribute to the outcome. By combining the power of a large 43,568-person discovery data set and stringent replication by the well phenotyped Nordic data sets, we identified and replicated six maternal genomic loci robustly associated with gestational length and three of them also associated with preterm birth with genomewide significance (P<5E-8) in the joint analysis.

The top four replicating genomewide significant SNPs for gestational length are in biologically plausible genes. *EBF1* (early B-cell factor 1), also achieving genomewide significance for preterm birth, has been demonstrated to be essential for normal B cell development,^51^ and recent genomewide association studies have implicated it in control of blood pressure,^52,53^ carotid artery intima media thickness,^54^ hypospadias,^35^ and metabolic risk.^55^ Whether *EBF1* confers its effect on birth timing through pregnancy-specific mechanisms, or by contributing to more general cardiovascular or metabolic traits that influence gestation remains to be determined. In addition, the association between this locus and gestational length may explain the effect of this locus on birth weight reported by Horikoshi et al.^43^

*EEFSEC* (eukaryotic elongation factor, selenocysteine tRNA-specific), also genomewide significant for both gestational length and preterm birth risk, participates in the incorporation of selenocysteine into selenoproteins. Selenoproteins, such as the glutathione peroxidases and thioredoxin reductases, serve critical cellular homeostatic functions in maintaining redox status and antioxidant defenses, as well as modulating inflammatory responses.^56^ These physiologic functions have previously been linked to the parturition process and preterm birth.^5,57,58^ Moreover, the SNPs we identified in *EEFSEC* are in high LD with SNPs that have previously been associated with age of onset of menarche, expression quantitative trait loci (eQTLs) for *EEFSEC* abundance, risk of prostate cancer^34^ and hypospadias.^35^ Intriguingly, the identification of the selenocysteine pathway suggests the potential benefit for further evaluating the role of maternal selenium micronutrient status on prematurity risk. While a recent Cochrane review of multiple micronutrient supplementation did not demonstrate a reduction in preterm birth risk,^59^ the studies included for analysis did not all utilize selenium as part of their supplement. Indeed, a recent evaluation of maternal serum selenium concentration in early pregnancy demonstrated reduced selenium concentration in association with preterm birth,^60^ and, while of multi-factorial etiology, the country with the highest global preterm birth risk, Malawi,^61^ demonstrates a high frequency of selenium-deficiency.^62^

*AGTR2* (angiotensin II receptor, type 2), the coding gene nearest to a group of X chromosome SNPs achieving genomewide significance in the gestational length analysis, had suggestive association with preterm birth discovery stage, and genomewide significance for preterm birth in the joint analysis with robust association in the Nordic replication. *AGTR2* has been suggested to play a role in modulating uteroplacental circulation, and harbors variants that may contribute to the risk of preeclampsia.^63,64^ The involvement of the renin-angiotensin system in blood flow at the maternal-fetal interface and oxidative stress, interacting with the selenoprotein glutathione perioxidase, a target for *EEFSEC*, is a potential shared mechanism for these genes in spontaneous preterm birth.^65^ It is unlikely that our association detects risk for preeclampsia rather than spontaneous preterm birth, because women with preeclampsia as a reason for their delivery were excluded in the Nordic studies, and were removed from the 23andMe discovery data set if medical indications for delivery were reported. 

The final gene locus achieving genomewide significance in the discovery stage for gestational length was *WNT4* (wingless-type MMTV integration site family member 4), with strong replication in the Nordic populations. *WNT4* mutations have been found in women with Mullerian duct abnormalities, primary amenorrhea, and hyperandrogenism,^66^ and common variants in *WNT4*, in high LD with our index SNPs, are associated with risk for endometriosis^38^, ovarian cancer^39^ and bone mineral density.^40^ Our analysis indicates that the minor allele (T) of the putative causative variant rs3820282 in the Nordic populations is associated with longer gestational length and is protective for preterm birth. rs3820282 is located in an active chromatin domain in the first intron of *WNT4*, and the T allele generates a strong ESR1 binding site, and as such likely alters estrogen-based regulation of *WNT4* and/or adjacent genes. The role of estrogen signaling as the functional consequence of the polymorphism is further supported by the association of the same region with endometriosis and ovarian cancer, both hormone-responsive disorders. Further, the parallel of the spectrum of disorders associated with the WNT locus mirrors that of ARID1A, also critical for endometrial function early in pregnancy, with loss of function variants causing atypical endometriosis and ovarian cancer, and enhanced estrogen activity. ^67,68^ Lastly, the population frequencies for endometriosis (Asian>European>African ancestry) trend in the same direction as does the T allele for rs3820282 (EAS 0.49 > EUR 0.14 > AFR 0.01 based on 1000 Genomes).^69,70^ *WNT4* did not achieve genomewide significance or suggestive association in the preterm birth risk dichotomous trait analysis, suggesting its role may be largely exerted near term gestation. 

*ADCY5* (adenylyl cyclase type 5) and *RAP2C* (member of the RAS oncogene family) achieved near genomewide significance in the discovery stage and were successfully replicated (Table 1). SNPs at the *ADCY5* locus have been reported to be associated with birth weight^41^ and type 2 diabetes;^42^ however, none of them were in close LD with the SNPs showing significant association with gestational length, suggesting shared mechanisms coordinating the duration of gestation with growth. The SNP rs2747022 in the *RAP2C* region (in gene *FRMD7*) was previously reported to be associated with spontaneous preterm delivery in Danish/Norwegian studies (the samples used in this previous study overlap with our replication samples).^22^ Several additional loci (*BOLA3*, *TEKT3* and *TGFB1*), while showing marginal evidence of replication, remain suggestive and await the addition of further studies for analysis. 

The primary limitation of our study centers on the characteristics of our study data sets. The gestational length information of the 23andMe samples was self-reported, and 3.2% of women in the preterm group did not respond as to whether the labor and delivery was spontaneous or medically indicated. In the term group, we were not able to unambiguously determine spontaneous from medically-indicated births. Despite these limitations, we included these samples in order to dramatically increase the sample size of the discovery stage, recognizing that our replication data sets would be more precisely phenotyped for spontaneous preterm birth. A previous study suggested that approximately 90% of mother-reported gestational lengths agreed with their associated medical records.^71^ In addition, other than maternal age and ancestry inferred by genotypes, other covariates were not available for the 23andMe samples. 

Our study demonstrates the utility of combining large samples with self-reported phenotyping with more modestly sized but precisely phenotyped replication studies to reveal maternal loci associated with gestational length and preterm birth. With this foundation, future expansion of maternal and fetal genotyped samples associated with gestational length information is anticipated to further refine our understanding of human pregnancy, risk for adverse pregnancy outcomes, and targeting of new preventive strategies for preterm birth. As the National Institutes of Health expand “Precision Medicine” initiatives in the years ahead, we would argue that the optimal time to advance human health is before and during pregnancy. Our work suggests that integration of genomic information on women, and likely their offspring, with birth timing, may allow development of new options for preventative and therapeutic measures.

## Supplementary Material

Supplementary Appendix
