# Supplementary material for: Genetic Associations with Gestational Length and Spontaneous Preterm Birth
Source: N Engl J Med. Author manuscript. (PMC5561422; doi:10.1056/NEJMoa1612665)
Supplement: Supplementary Appendix [file NEJMoa1612665-Muglia-Supplement.pdf]

Contents

Supplementary Text 3

Overview of study design 3

Discovery methods 3

Replication methods 5

Functional annotation of SNPs at significant loci 7

Other statistical and bioinformatic analyses 8

Functional analyses of the WNT4 locus 10

Supplementary Figures 13

Figure S1. Histogram of self-reported gestational age in the 23andMe data set 13

Figure S2. Distribution of gestational length in the three Nordic data sets (Top:FIN; Middle:MoBa; Bottom: DNBC) 14

Figure S3. QQ plot of GWA results (discovery phase) 15

Figure S4. Correlation between effect sizes estimated from mothers and infants (replication phase) 16

Figure S5. Regional plots of genomic loci associated with gestational age 17

Figure S6. Regional plots of genomic loci associated with preterm birth 24

Figure S7. Variance explained in the replication studies using associated SNPs 29

Figure S8. Manhattan plot of gene-wise association *P* values 30

Figure S9. Gene set enrichment analysis showing enrichment of association signals in genes implicated in pregnancy related biological terms 31

Supplementary Tables 32

Table S1. Study datasets 32

Table S2. Phenotype data of discovery samples (23andMe) 33

Table S3. Influences of co-variates in discovery samples 34

Table S4. Influence of covariates on gestational age (replication studies) 35

Table S5. Suggestive GWA regions (discovery phase) 36

Table S6. Association results obtained from “spontaneous only” and the full discovery data set. ... 37

Table S7. Discovery and replication of GWA loci associated with gestational age 38

Table S8. Discovery and replication of GWA loci associated with preterm birth 40

Table S9. Associations with known GWA SNPs 41

Table S10. eQTLs in GWA loci associated with gestational length 42
Table S11. eQTLs in GWA loci associated with preterm birth 44
Table S12. Replication of GWA loci associated with gestational age using infant samples 45
Table S13. Replication of GWA loci associated with preterm birth using infant samples.................. 47
Table S14. Comparison of effect sizes estimated from mothers and infants.................................... 48
Table S15. Joint maternal and fetal genetic association analysis in mother/infant pairs 49
Table S16. Conditional association tests of replicated loci.. 50
Table S17. Variance explained by all common SNPs (MAF > 0.01) estimated using GCTA 51
Table S18. Variance explained in the replication studies using GWA SNPs identified from discovery data set (23andMe).. 52
Table S19. Difference in gestational length between individuals with high (top quartile) and low (bottom quartile) genetic scores 53
Supplementary References 54

Supplementary Text

Overview of study design

We performed a two-stage genomewide association study to discover and replicate associations of genetic loci with gestational age as a continuous trait and preterm birth as a dichotomous trait. In the discovery stage, we performed genomewide association analysis in more than 40 thousands unrelated European women identified among 23andMe's research participants. In the replication stage, the most significant findings from the discovery stage were tested in 8,643 mothers from three independent birth studies from Nordic countries (Finland, Denmark, and Norway).

Discovery methods

Participants in the 23andMe data set were customers of 23andMe who consented to participate in research and answered survey questions online. Women of European ancestry who reported live singleton birth with gestational length information (in weeks) were included in the analysis. Women who reported a medical indication for their delivery were excluded from the study as we sought mechanisms for spontaneous birth; those who did not specify a choice of spontaneous or medically indicated delivery on the survey were retained to optimize sample size. Preterm birth status was assigned to individuals based on dichotomization of their self-reported gestational age (preterm term< 37 weeks; normal term ≥ 37 weeks). The under-representation of women reporting 39 weeks of gestation reflects the survey structure. Women were first asked whether their delivery was "more than a week before my due date", "within a week of my due date", or "more than a week after my due date". Women who answered "within a week of my due date" were assigned a gestational age of 40 weeks. Women who answered other than "within a week of my due date" were asked a follow-up question to determine weeks of gestation (Fig. S1).

DNA extraction and genotyping were performed on saliva samples by National Genetics Institute (NGI), a CLIA licensed clinical laboratory and a subsidiary of Laboratory Corporation of America. Samples were genotyped on one of four genotyping platforms. The V1 and V2 platforms were variants of the Illumina HumanHap550+ BeadChip, including approximately 25,000 custom SNPs selected by 23andMe, with a total of approximately 560,000 SNPs. The V3 platform was based on the Illumina OmniExpress+ BeadChip, with custom content to improve the overlap with the V2 array, with a total of approximately 950,000 SNPs. The V4

platform is a fully custom array, including a lower redundancy subset of V2 and V3 SNPs with additional coverage of lower-frequency coding variation, and about 570,000 SNPs. Samples were required to have a call rate of 98.5% or higher to be included in the study.

To minimize the effects of population stratification, our analyses were restricted to women who have >97% European ancestry, as determined through an analysis of local ancestry.[1] The reference population data is derived from public datasets (the Human Genome Diversity Project, HapMap, and 1000 Genomes), as well as 23andMe participants who reported having four grandparents from the same country. A maximal set of unrelated individuals was chosen for each analysis using a segmental identity-by-descent (IBD) estimation algorithm.[2] Individuals were defined as related if they shared more than 700 cM IBD, including regions where the two individuals share either one or both genomic segments identical-by-descent.

Participant genotype data were imputed against the September 2013 release of 1000 Genomes Phase1 reference haplotypes. The 23andMe participants were phased using an internally developed phasing tool, Finch, which implements the Beagle haplotype graph-based phasing algorithm,[3] modified to separate the haplotype graph construction and phasing steps.

In preparation for imputation, SNPs deviated from Hardy-Weinberg equilibrium ($P < 1\times10^{-20}$), with low call rate (< 95%), or with large allele frequency discrepancies compared to European 1000 Genomes reference data were excluded. Frequency discrepancies were identified by computing a 2x2 table of allele counts for European 1000 Genomes samples and 2000 randomly sampled 23andMe participants with European ancestry, and identifying SNPs with a chi squared test ($P < 1\times10^{-15}$). We imputed phased haplotypes against all-ethnicity 1000 Genomes haplotypes (excluding monomorphic and singleton sites) using Minimac2,[4] using 5 rounds and 200 states for parameter estimation.

Single-marker tests for genetic association with gestational age and preterm birth were performed by linear regression and logistic regression, respectively, using imputed allelic dosage data assuming additive allelic effects. Maternal age and the top five principal components to account for residual population structure were included as covariates. The association test *P* values were computed using a likelihood ratio test.

We clustered SNPs into association regions (or loci) based on their *P* values and physical proximity. Specifically, we defined association regions by firstly identifying SNPs with association $P < 1\times10^{-4}$, then grouping these into a region if they were adjacent to each other (<250kb). The SNP with smallest *P* value (leading SNP) within each region was chosen as the

index SNP. Regions that achieved genomewide suggestive significance ($P < 1\times10^{-6}$) were tested for replication in the replication stage. The significant SNPs ($P < 1\times10^{-6}$) and their close proxies ($r^2 > 0.8$) with in each putative region were examined in the replication stage.

To confirm the robustness of the association signals, we conducted association tests in a subset of discovery samples who explicitly checked "spontaneous delivery" in the questionnaire (excluding those who did not specify a choice of spontaneous or medically indicated delivery) and compared the results with those obtained from the full discovery data set (Table S6).

Replication methods

We used phenotype and genomewide SNP data of 8,643 mothers from birth studies collected from three Nordic countries to replicate the discovery stage findings (Table S1).

The Finnish dataset (FIN) was collected for a genetic study of preterm birth.[5] Briefly, whole blood samples were collected from more than 800 mother/child pairs from the Helsinki (southern Finland) University Hospitals between 2004 and 2014. All of these studied samples are of Finnish descent. Crown-rump length at the first ultrasound screening between 10+ and 13 weeks was used to determine the gestational age. The study was approved by the Ethics Committee of Oulu University Hospital and that of Helsinki University Central Hospital. Written informed consent was given by all participants.

The Mother Child dataset of Norway (MoBa) is a nationwide Norwegian pregnancy study administered by the Norwegian Institute of Public Health. The study includes more than 114,000 children, 95,000 mothers and 75,000 fathers recruited from 1999 through 2008.[6] Gestational age was estimated by ultrasound at gestational weeks 17–19. In the few cases without ultrasound dating, gestational age was estimated using the date of the last menstrual period. For the current study, we used the mother-child pairs that were selected from Version 4 of the MoBa dataset, which included a total of 71,669 pregnancies.[7] Singleton live-born spontaneous pregnancies with mothers in the age group 20–34 years were selected. Pregnancies involving pre-existing medical conditions, pregnancies with complications as well as pregnancies conceived by in vitro fertilization, were excluded from the study. Random sampling was done from two gestational age ranges 154-258 days (cases) and 273–286 days (controls). In total, blood samples from 3,121 mothers and children were genotyped.[7] 1,834 mothers and 1,143 infants that passed QC were included in the analysis. All parents gave informed, written consent. The study was approved by The Regional Committee for Medical Research Ethics in South-Eastern, Norway.

The Danish National Birth Cohort (DNBC) followed over 100,000 pregnancies between 1996 and 2003 with extensive epidemiologic data on health outcomes in both mother and child.[8] The current study used genotype data generated in two genomewide association studies. One was a study of preterm delivery[9] and the second was a study of obesity,[10,11] totally including 5,921 mothers and 2,130 infants after QC. Gestational age in this dataset was determined by a consensus algorithm combining all available information from multiple sources: self-reported date of last menstrual period, self-reported delivery date, and gestational age at birth registered in the Medical Birth Register and the National Patient Register. The study protocol was approved by the Danish Scientific Ethical Committee and the Danish Data Protection Agency.

All the preterm births included in the Nordic studies were spontaneous. Obstetrical induction of labor, placental abnormalities, preeclampsia, congenital malformations and multiple births were excluded. Pregnancies involving pre-existing medical conditions known to be associated with preterm birth, pregnancies with complications were also excluded. In the MoBa dataset only pregnancies with mothers in the age group of 20-34 years were selected.

Genotyping was conducted on DNA extracted from blood using the various SNP arrays previously presented.[12] Specifically, for the FIN dataset, genotyping was conducted using Affymetrix 6.0 (Affymetrix, California, United States) and various other Illumina arrays (Illumina, California, United States). For the Affymetrix SNP Array 6.0, genotype calls were determined using the CRLMM algorithm [13,14] among chips that passed the vendor-suggested QC (Contrast QC > 0.4). For the Illumina chips, the genotype calling was conducted using Illumina's genotyping module v1.94 in the GenomeStudio v2011.1. The samples from the MoBa dataset were genotyped using the Illumina Human660W-Quadv1_A bead chip (Illumina Inc.) and the genotype calls were determined using CRLMM algorithm. The DNBC samples were genotyped using Human660W-Quad (preterm birth study) and Human610-Quad (obesity study) Bead Arrays from Illumina. Genotype calls were determined using GenomeStudio software (Illumina).

Similar quality control (QC) procedures were used across the three Nordic studies. Briefly, individuals with incorrect sex assignment, excessive heterozygosity or low call rate (<98%) were excluded from further analysis. Incorrect mother-child relationships and cryptic relatedness were detected by identity-by-descent (IBD) sharing estimated from genomewide SNPs. Non-European samples were identified and excluded using principal components analysis (PCA) anchored with 1000 Genomes reference samples. At marker level, SNPs with low call rate (<98%), low minor allele frequency (<0.03) or significant deviation from Hardy-Weinberg Equilibrium ($P < 1\times10^{-4}$) were excluded.

We conducted genomewide imputation. A standard two-step imputation procedure was followed: the genotype data of the mother and infants was first pre-phased together using Shapeit2 software[15] and then the estimated haplotypes were used to impute untyped SNPs using the reference haplotypes extracted from the Phase I 1000 Genomes Project[16] by either Minimac2[4] (FIN and MoBa) or Impute2[17] (DNBC).

Single-marker genetic association tests were conducted in individual replication studies separately, using regression methods and imputation dosage data similar to the discovery stage. In the replication stage, infant gender was included as covariate. Genotypic association tests (d.f. = 2) were also performed to examine possible dominance effect. The test results (inflation adjusted estimates of effect sizes and *P*-values) from the three Nordic studies were combined by fixed effect meta-analysis using the inverse-variance method. In addition, we performed the same association tests in infant samples to distinguish the maternal or fetal origin of the observed significant associations.

The replication-stage results were generated using meta-analysis in the three Nordic studies. All discovery-stage SNPs with $P < 1\times10^{-6}$ together with their LD-proxies ($r^2 > 0.8$) were examined in the replication stage. We estimated the effective number of independent SNPs tested in each discovery region[18] using the program simpleM[19] that adjusts for high inter-marker LD. The marker-wise significance thresholds for replication were determined using Bonferroni adjustment: dividing the desired family-wise error rate (0.05) by the effective number of independent SNPs in a discovery region and the total number of discovery regions. Replication-stage *P* values passing this threshold and having the same direction of effect as in discovery stage were regarded as evidence of successful replication. Variants were considered genomewide significant if they were significantly replicated and had a combined discovery and replication *P* value less than 5×10^{-8}.

Functional annotation of SNPs at significant loci

We did functional annotation of SNPs at loci with genomewide suggestive significance (discovery $P < 1\times10^{-6}$). Within each of those putative regions, we first identified genic SNPs that might have functional consequences (i.e. nonsense, missense and splicing SNPs) by searching dbSNP (build 149) and then examined whether these “functional” SNPs are in close LD with the any of the SNPs showing significant association with gestational length or preterm birth with discovery $P < 1\times10^{-6}$. We also checked whether the SNPs associated with gestational length or

preterm birth overlap or are in close LD with previously reported genomewide association SNPs in GWAS catalog[20] (r2017-01-09).

We used the Genotype-Tissue Expression (GTEx)[21] database to examine the potential downstream regulatory effects of the SNPs associated with gestational length or preterm birth. The significant SNPs (discovery $P < 1\times10^{-6}$) and their close proxies ($r^2 > 0.8$) with in each putative region were searched against the GTEx dataset for their potential regulatory effect on gene expression level in different tissues.

Other statistical and bioinformatic analyses

To examine if there are multiple independent variants influencing the trait at a locus, we performed an approximate conditional and joint multiple-SNP (COJO) analysis[22] using the summary statistics of the discovery stage. This method takes linkage disequilibrium (LD) between SNPs into account and adopts a stepwise selection procedure to select SNPs on the basis of conditional *P* values[23] and estimate the joint effects of all selected SNPs[22]. The genotype data of the 1000 Genome European samples were used to estimate the LD between SNPs. This analysis revealed a number of loci with $P < 1\times10^{-4}$ at a secondary SNP after conditioning on the index SNP (Table S16, Figure S5 and S6). At the *EBF1* locus the secondary signals were significant even at a Bonferroni-corrected significance threshold of $P < 2.26\times10^{-5}$, based upon genome coverage of 0.22% (6.6 Mb) by the tested loci. It should be noted that this analysis is exploratory and the existence of secondary association signal needs to be confirmed in large samples with regional genotype data obtained directly by sequencing.

In the replication dataset, we estimated the fraction of phenotype variance explained by SNPs. We first estimated the fractions of phenotype variance in gestational age and preterm birth explained by all SNPs with MAF>0.01 in individual replication studies using GCTA[24] and then combined the results across the three replication using the inverse-variance method. The results (Table S17) indicated that, in our replication data sets, the estimated variance explained by all common SNPs (MAF > 0.01) in mothers were ~16% and ~21% for gestational length and preterm birth (liability scale), respectively.

We also examined the percentage of variance explained by the genomewide association SNPs identified from the discovery stage. At different significance levels ($\alpha = 1\times10^{-6} – 5\times10^{-5}$), sets of index SNPs of the putative associated loci were selected to construct the weighted genetic score. The variance explained in the replication samples was then calculated based on a linear regression model using the constructed genetic score as predictor and the covariates

(maternal age and infant gender) adjusted phenotype (gestational age or preterm birth status) as outcome. We found that, in our replication samples, the genetic score that was constructed based on a dozen to one hundred leading SNPs selected at different significance thresholds explained ≤ 1% of the observed variance in gestational length (Table S18 and Figure S7). The difference in gestational length was approximately 5 days (95% CI: 3.2, 7.0) between the top and bottom genetic quartile based on the genetic score constructed using the 12 index SNPs with discovery P-value < $1{\times}10^{-6}$ (Table S19).

We also performed gene-centric association and gene-set enrichment analyses using MAGMA.[25] First, we used PubMed abstract database to create 28 sets of genes related to various keywords: terms related to pregnancy (e.g. *myometrium, preterm*) and control terms (e.g. *liver, sleep*). For each keyword, all PubMed abstracts containing that keyword and the words "gene", "genes", "genome", "genomic", "genetic", "GWAS" were retrieved. Abstracts mentioning certain pregnancy-complicating diseases (e.g. *diabetes*, *hypertension*) were excluded, as well as abstracts mentioning other animal species, as pregnancy mechanisms are likely to differ in humans. Remaining abstracts were scanned for any HGNC gene names, excluding symbols that match certain common abbreviations (e.g. *DNA, PCR*). All gene names detected this way comprise the gene-set for that particular keyword.

MAGMA[25] was then used to summarize SNP-level *P* values into gene- and gene-set-level *P* values. Gene-level *P* values represent the mean association strength of all SNPs located within a particular gene or 50 kb distance from it (see "mean" combination method in MAGMA documentation). Gene-set-level *P* values are obtained by comparing the *P* values of genes that belong to a particular gene-set vs. *P* values of all other genes, while taking into account gene size and density (see "competitive" gene-set analysis in MAGMA documentation). Since the gene-sets constructed here reflect current knowledge of the genetic factors concerning each keyword, we expect obstetrics-related gene-sets to show significant *P* values if the genomewide association study indeed worked. Similarly, the control keywords are expected to generate non-significant *P* values.

The gene-centric association using MAGMA[25] showed (Figure S8) a similar pattern with the marker-wise analysis (Figure 1) but with the most significant gene-wise *P*-value observed at the *EEFSEC* gene. Gene-set enrichment analysis demonstrated that the association signals were highly enriched in genes that have been implicated in pregnancy-related biological concepts such as "preterm," "endometrium," "parturition," and "uterus" (Figure S9).

Functional analyses of the WNT4 locus

We conducted functional analysis of the *WNT4* locus, given its plausible functional relevance in pregnancy. First we examined the expression level of *WNT4* in human endometrial stromal cells, before and after decidualization using mRNA-seq technology. Two primary human endometrial stromal fibroblast (ESF) lines (HsESC_217S and HsESC_218S) were obtained from the Hugh Taylor/Clare Flannery labs in the Department of Obstetrics, Gynecology, and Reproductive Sciences at Yale University. HsESC_217S and HsESC_218S were derived from two separate patients each undergoing a polypectomy surgical procedure on days 3 and 9 of the menstrual cycle, respectively. The cells were grown in DMEM supplemented with 10% charcoal-stripped fetal bovine serum (Gemini), 0.1% ITS (Corning), and 1% antibiotic/antimycotic (ABAM; GIBCO). To induce decidualization, the cells were treated with 0.5mM 8-Br-cAMP (Sigma) and 0.5mM of the progesterone analog, medroxy-progesterone acetate (MPA), for 48 hours in DMEM supplemented with 2% fetal bovine serum (gemini). Cells were examined for markers of ESF cells and markers of normal decidualization.

Total RNA was extracted using the RNeasy Mini kit (Qiagen) followed by on-column DNase I treatment. Total RNA quality was assayed with the Bioanalyzer 2100 (Agilent). RIN values for all samples were above 9.5. RNA samples were then sequenced using the Illumina HiSeq2500. A minimum of 36 million paired end reads were obtained for each sample. Reads were aligned to the human (GRCh37.69) cDNA builds at Ensembl with TopHat2. Reads mapping to each gene feature were counted with HTSeq, and the counts were normalized as transcripts per million (TPM).[26] Three independent samples of each of the primary cell cultures were taken and the expression values used here are the averages for every gene of the three transcriptomes.

We predicted specific transcription factors whose binding might be altered by gestational length-associated variants that localize in the *WNT4* locus using the CisBP web server[27] and confirmed the presence of H3K4me3 marks overlapping rs3820282 in a telomerase immortalized endometrial stromal cell line (ATCC CRL-4003) using ChIP-seq[28] technology. Specifically, ChIP-seq was performed using anti-H3K4me3 (Invitrogen, 49-1005) antibody following a previously described protocol.[29] Library preparation and high-throughput sequencing were carried out on the Illumina Genome Analyzer II platform by following the protocol suggested by Illumina for sequencing chromosomal DNA. Two replicates were sequenced at 1x75 bp by the Yale Center for Genome Analysis. Sequence reads were aligned to the human reference genome (hg19) using the ultra-fast short DNA sequence aligner Bowtie2.[30,31] The sequencing depth was 32 million reads per replicate for all input and ChIP samples from

decidualized ESF and 52 million reads per replicate for all input and ChIP samples from ESF. The average alignment rate was 75% for samples from decidualized ESF and 85% for samples from ESF. Only uniquely aligned reads were used for analysis. Visualization of the reads at functional genomic regions was obtained using D-peaks.[32]

We studied the presence of H3K4me3 marks and open chromatin domain by ATAC-seq. ATAC-seq sample preparation was adapted from [33] (Buenrostro, Giresi et al. 2013). Endometrial stromal cells were harvested by trypsinization. After counting, $5x10^4$ cells were washed with 50µl cold PBS followed by 50µl cold lysis buffer (10mM Tris-HCl, pH 7.4; 10mM NaCl; 3mM $MgCl_2$; 0.1% NP40) to prepare nuclei. Nuclei were resuspended in 50µl transposase reaction mix (from Illumina Nextera DNA Sample Preparation kit, Cat. FC-121-1030: 25µl 2x TD Buffer; 2.5µl Transposase; 22.5µl nuclease-free water) and incubated for 30 min at 37°C to allow transposition of sequencing adapters into open chromatin. Transposed sample ("tagmented" DNA) was purified using Qiagen MinElute Reaction Clean-up kit (Cat. 28204), and PCR amplified for an average of 10 cycles using NEBNext High-Fidelity 2x PCR Master Mix (NEB Cat. M0541S) and Nextera Index Primers (Illumina Cat. FC-121-1011). To avoid amplification biases arising from limiting conditions, the optimum number of PCR cycles for each sample was determined as follows: the PCR reaction was stopped at 5 cycles; an aliquot of the reaction was used as the template for qPCR reaction with the same reaction conditions but with the addition of SYBR Green I dye; the cycle at which the rate of reaction begins to drop after hitting a peak was determined, and used to calculate the additional number of cycles necessary for library amplification. Amplified library was purified with Qiagen MinElute PCR Purification kit (Cat. 28004). The library was sequenced at Yale Center for Genome Analysis on Illumina HiSeq 2500 without multiplexing to get ~2x108 paired-end reads of 75bp.

We performed electrophoretic mobility shift assays (EMSA) to determine whether the polymorphism at the *WNT4* locus differentially affected estrogen receptor alpha binding. Human endometrial stromal fibroblasts (T HESC, obtained from Gill lab, Yale University, corresponding to ATCC CRL-4003) were cultured at 37˚C and 5% CO2 in DMEM supplemented with 10% fetal bovine serum (Gemini), 1% L-Glutamine, and 1% antibiotic/antimycotic (ABAM; GIBCO). To induce decidualization, the cells were treated for 72 hours with DMEM supplemented with 2% fetal bovine serum (Gemini), 0.5mM 8-Br-cAMP (Sigma), 1µM of the progesterone analog, medroxy-progesterone acetate (MPA), and 10nM estradiol (Sigma).

We prepared nuclear protein extracts based on Miller DE et al.[34] with minor modifications. Decidualized T HESC cells were trypsinized, washed twice with cold PBS, counted,

resuspended in PBS as 10^7 cells/ml, then centrifuged 3300xg for 2min, 4°C. PBS was removed and 10^7 cells were resuspended in 400µl CE buffer (10mM HEPES (pH7.9)), 10mM KCl, 0.1mM EDTA, 1mM dithiothreitol (DTT), and 1X HALT protease/phosphatase inhibitor (ThermoFisher)), then incubated on ice for 15 minutes. Cells were mixed with 25µl of 10% Nonidet P-40 by pipetting, and then nuclei were pelleted at maximum speed for 3min, 4°C. Nuclei were resuspended in 30µl NE buffer (20mM HEPES (pH7.9), 0.4M NaCl, 1mM EDTA, 1mM DTT, 1X protease/phosphatase inhibitor) by vortexing, incubated on ice for 10min, then centrifuged 3300x g for 2min, 4°C. Supernatants (nuclear proteins) were removed from pelleted debris and stored in small aliquots at -80°C. Protein concentration was determined by BCA.

Double-stranded IRDye700 5' end-labelled 41bp oligonucleotides, identical except for the nucleotide at rs3820282, being either "C" or "T", were obtained from IDT. The oligo sequence of the more common "C" allele is:

5' -IRDye700/GCCTGGAGGGCTCTGGCTGGCCACAATGACCTCATAGCAAC-3'.

The less common "T" allele oligo sequence is:

5' -IRDye700/GCCTGGAGGGCTCTGGCTGGTCACAATGACCTCATAGCAAC-3'.

DNA binding reaction buffer contained: 1x binding buffer, 1x DTT/Tw20, 1µg poly(dI-dC), 0.05% NP-40 (LI-COR EMSA buffer kit), 1mM zinc acetate and 10nM estradiol (Sigma). Binding reactions with nuclear extract (prepared as above) contained 12µg nuclear protein. Binding reactions with purified full-length bioactive human estrogen receptor alpha protein (ThermoFisher #A15674) contained 10ng purified protein. The HESC extracts were supplemented with purified ESR1 as, while endogenous ESR1 mRNA is present in these cells, its low abundance (approximately 5-6 TPM) limits the sensitivity of measuring endogenous estrogen receptor binding in this biologically relevant context. 50fmol fluorescent oligo DNAs were then added to the appropriate protein/binding mix and incubated for 20min at room temp. For supershift assays, 200ng rabbit monoclonal estrogen receptor alpha antibody (Cell Signaling #8644) was incubated with the nuclear lysate/binding buffer for 20min prior to addition of and incubation with oligo DNA. 1x orange loading dye (LI-COR kit) was added to samples, which were then resolved on (pre-cast, pre-run at 100V for 60min) 6% TBE gels (Novex, ThermoFisher) in 0.5x TBE buffer for 80min at 80V (room temp). Fluorescent bands were then imaged using a LI-COR chemiluminescent imaging system. EMSA experiments display representative panels of 2-3 replicates.

Supplementary Figures

Figure S1. Histogram of self-reported gestational age in the 23andMe data set

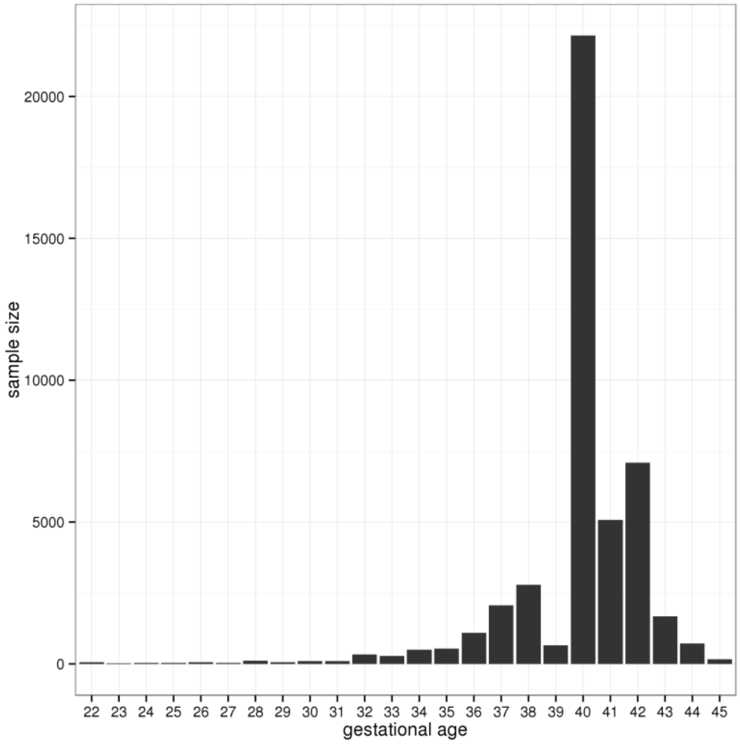

The under-representation of women reporting 39 weeks of gestation reflects the survey structure. Women were first asked whether their delivery was "more than a week before my due date", "within a week of my due date", or "more than a week after my due date". Women who answered "within a week of my due date" were assigned a gestational age of 40 weeks. Women who answered other than "within a week of my due date" were asked a follow-up question to determine weeks of gestation.

Figure S2. Distribution of gestational length in the three Nordic data sets (Top:FIN; Middle:MoBa; Bottom: DNBC)

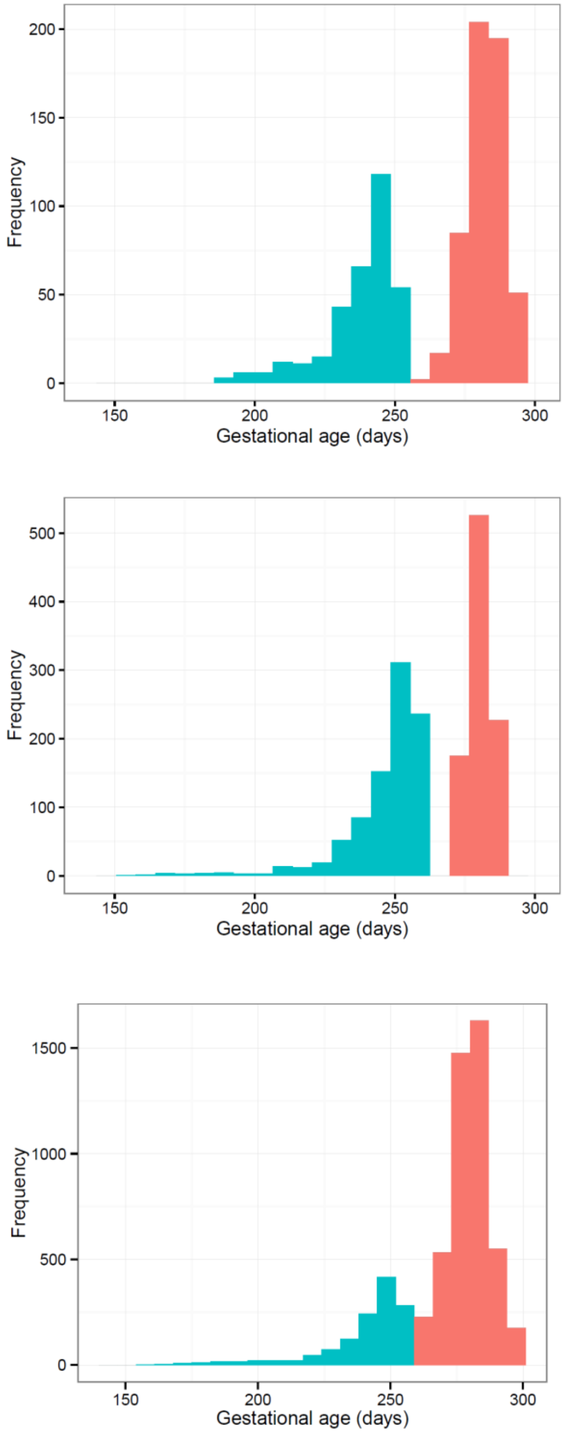

The histograms of gestational length in the three Nordic data sets. Gestational length in term and preterm samples were showed respectively in red and blue.

Figure S3. QQ plot of GWA results (discovery phase)

A. B.

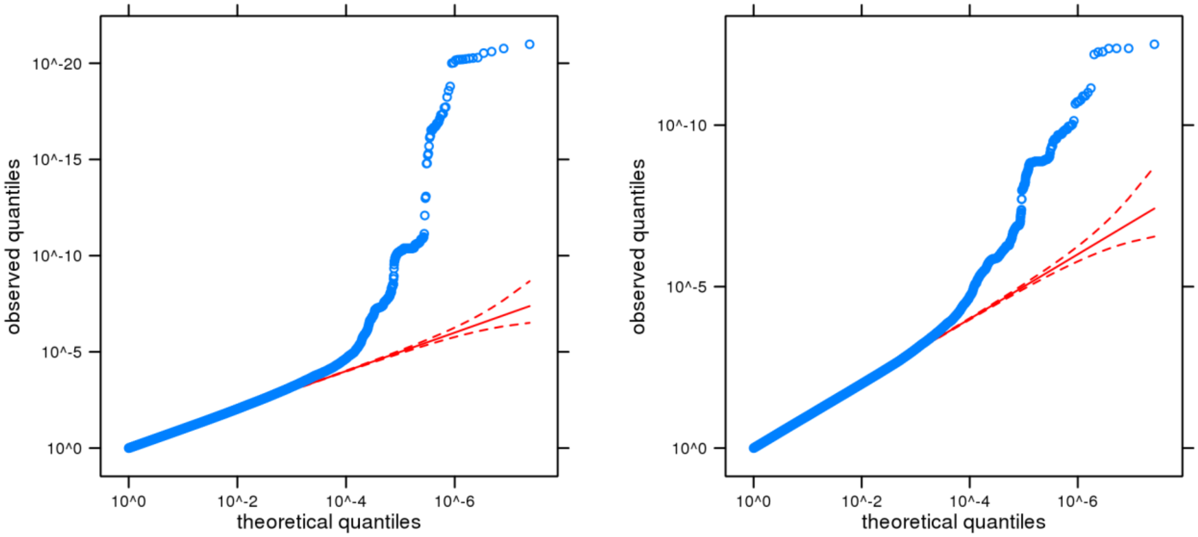

Lambda=1.038 Lambda=1.025

Figure S4. Correlation between effect sizes estimated from mothers and infants (replication phase)

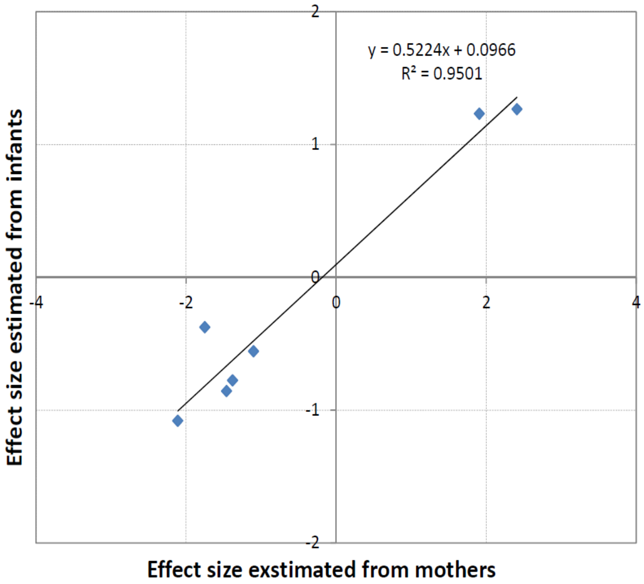

Figure S5. Regional plots of genomic loci associated with gestational age

Top: showing the primary association; Bottom: showing the secondary association after accounting for the primary association. The rs number and *P*-value of the most significant primary or secondary SNP were labeled on top of the SNP. r^2 between each SNP and the most significant SNP was color coded.

A. EBF1 locus

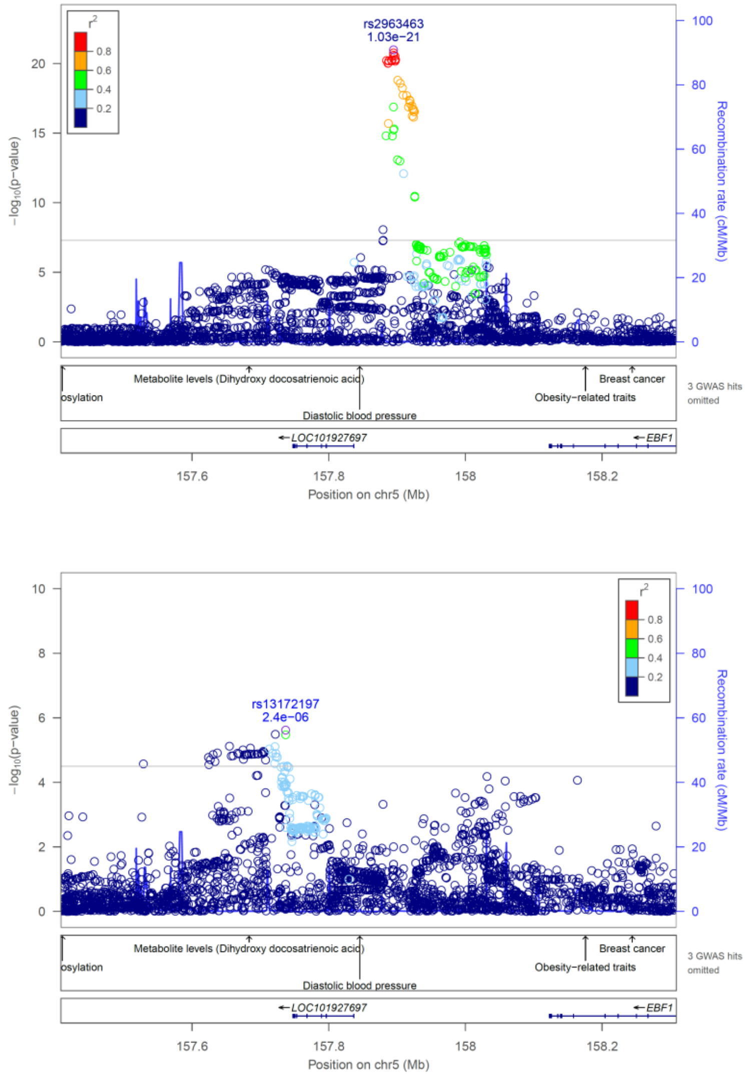

B. ***EEFSEC*** **locus**

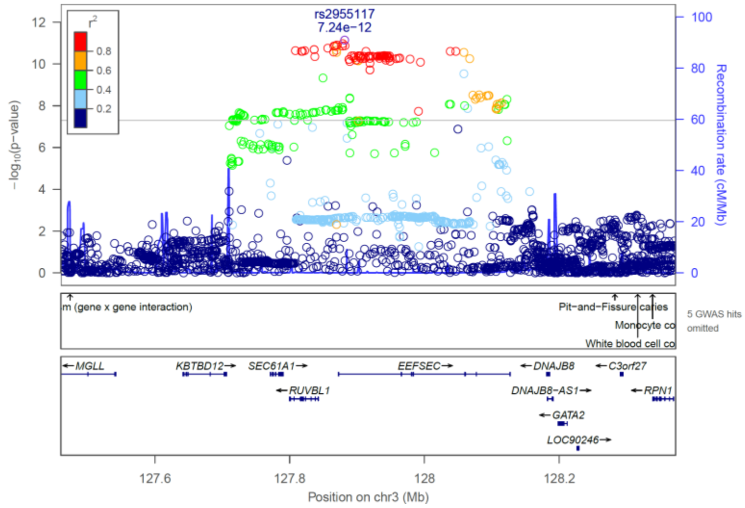

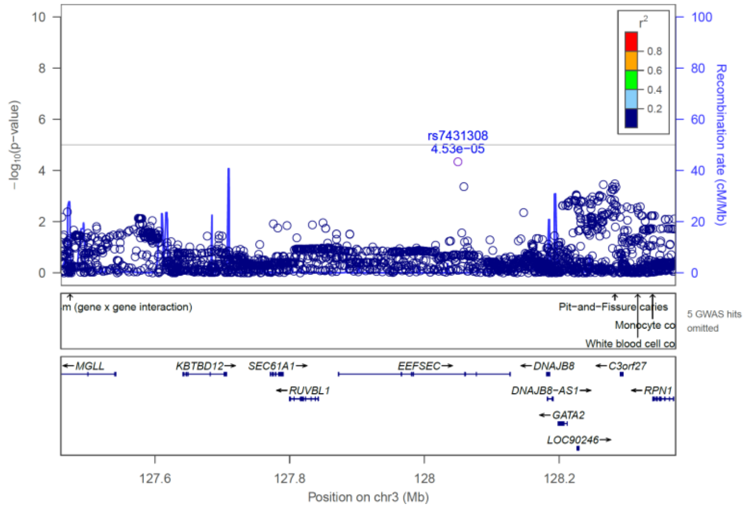

C. AGTR2 locus

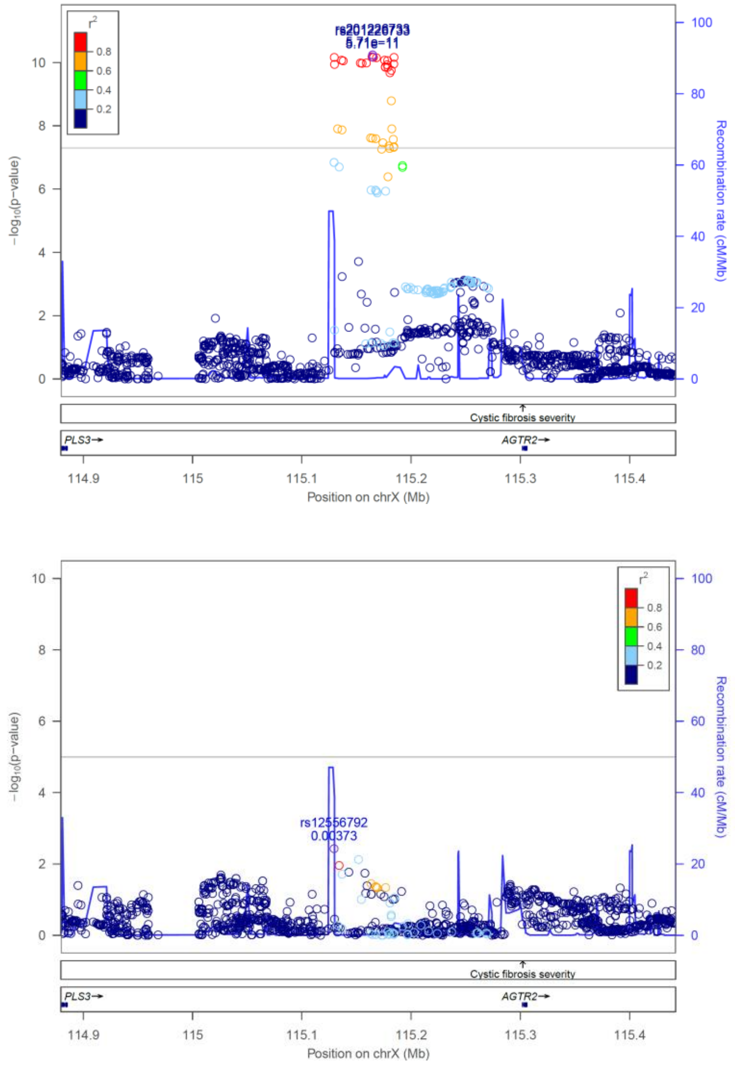

D. WNT4 locus

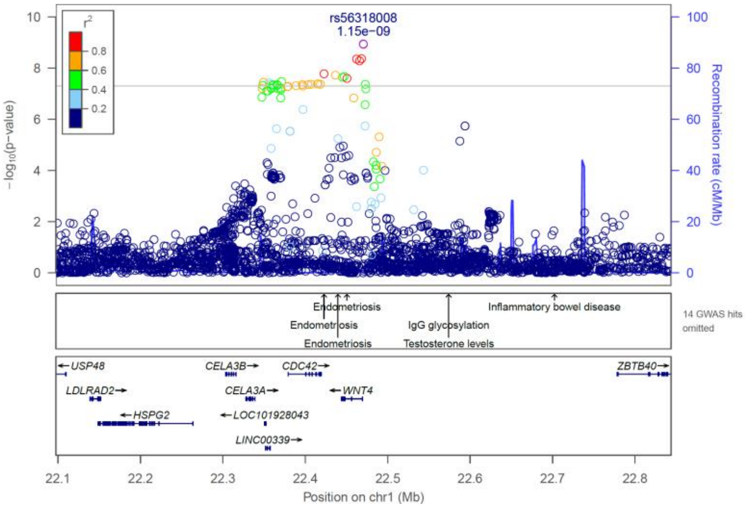
rs56318008
1.15e−09
r²
0.8
0.6
0.4
0.2
−log10(p−value)
Recombination rate (cM/Mb)
Endometriosis
Endometriosis
Endometriosis
IgG glycosylation
Testosterone levels
Inflammatory bowel disease
14 GWAS hits omitted
USP48
CELA3B
CDC42
ZBTB40
LDLRAD2
CELA3A
WNT4
HSPG2
LOC101928043
LINC00339
Position on chr1 (Mb)

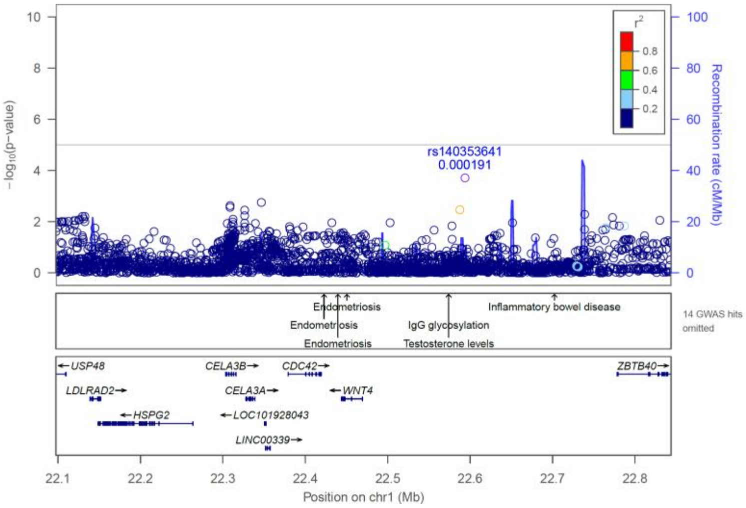
rs140353641
0.000191
r²
0.8
0.6
0.4
0.2
−log10(p−value)
Recombination rate (cM/Mb)
Endometriosis
Endometriosis
Endometriosis
IgG glycosylation
Testosterone levels
Inflammatory bowel disease
14 GWAS hits omitted
USP48
CELA3B
CDC42
ZBTB40
LDLRAD2
CELA3A
WNT4
HSPG2
LOC101928043
LINC00339
Position on chr1 (Mb)

E. *ADCY5* locus

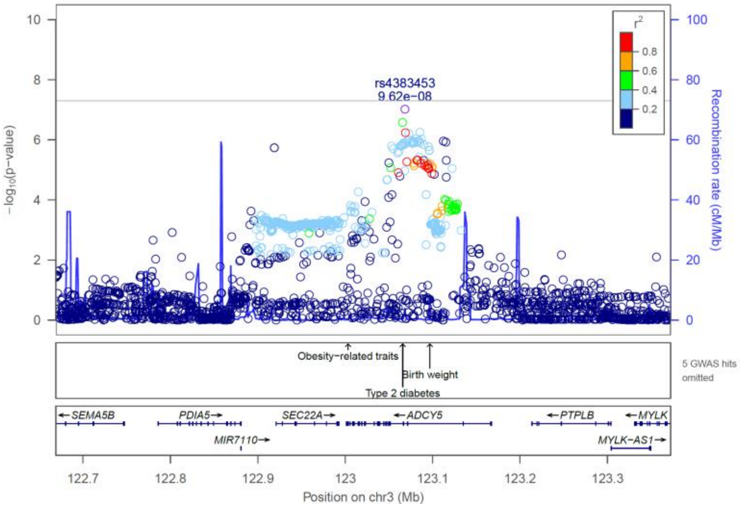

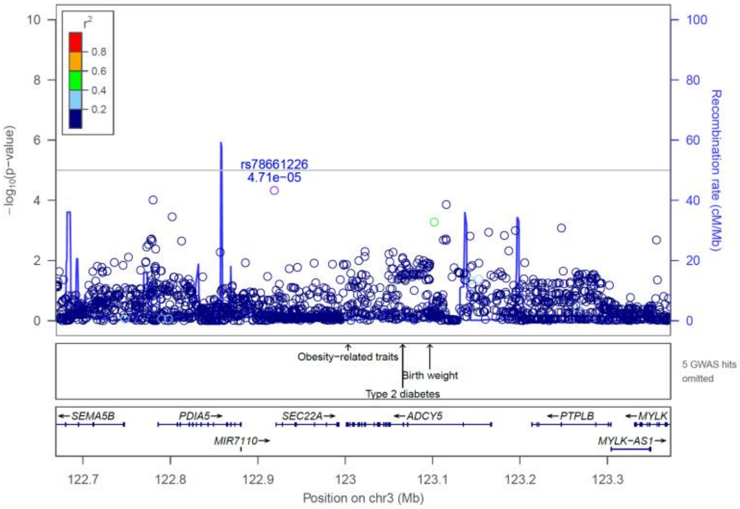

F. *RAP2C* locus

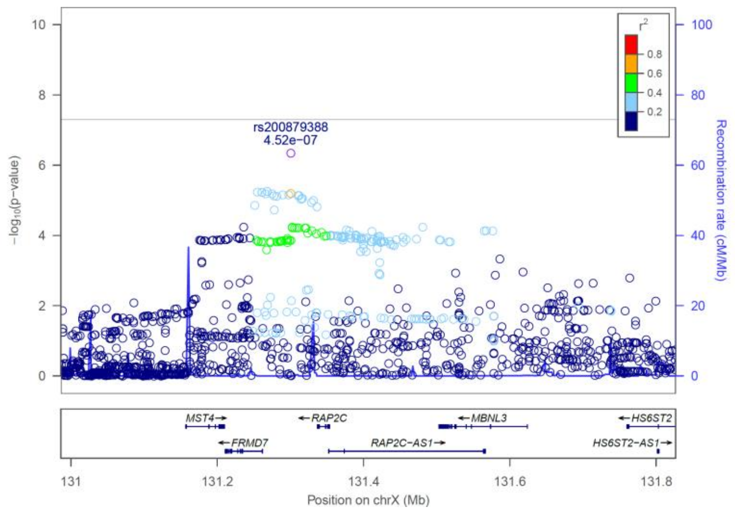

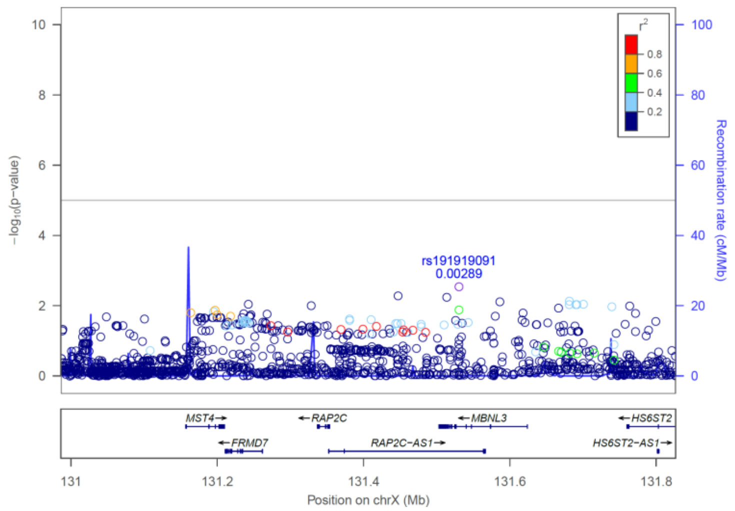

G. BOLA3 locus

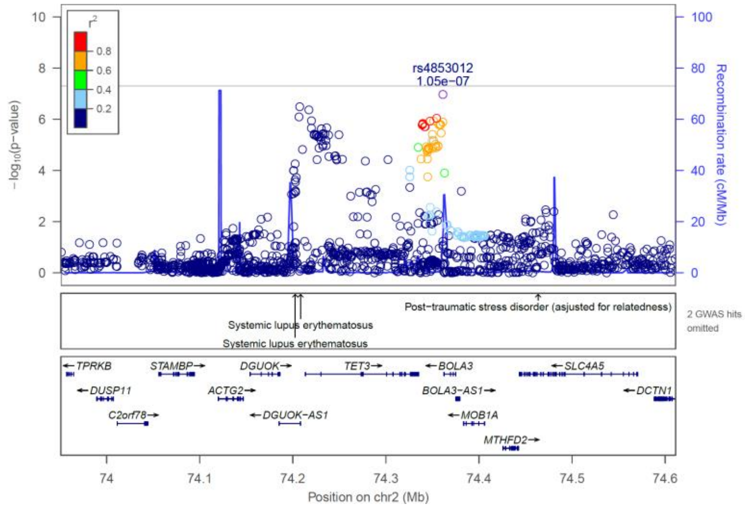

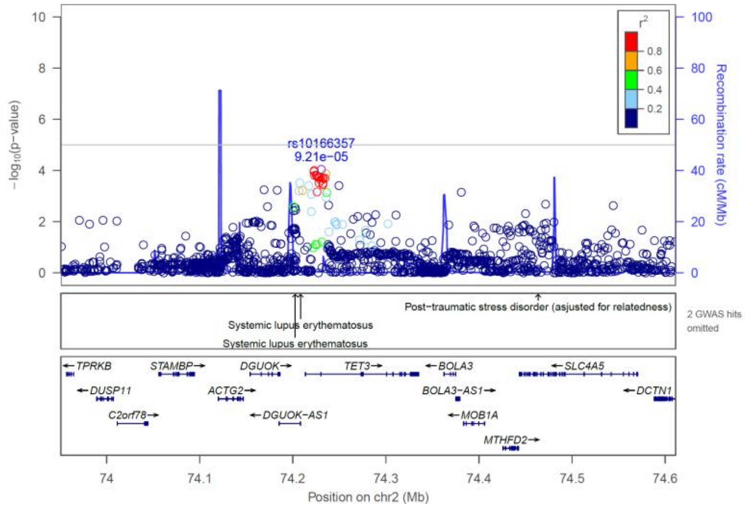

Figure S6. Regional plots of genomic loci associated with preterm birth

(top: showing the primary association; bottom: showing the secondary association after accounting for the primary association)

A. EBF1 locus

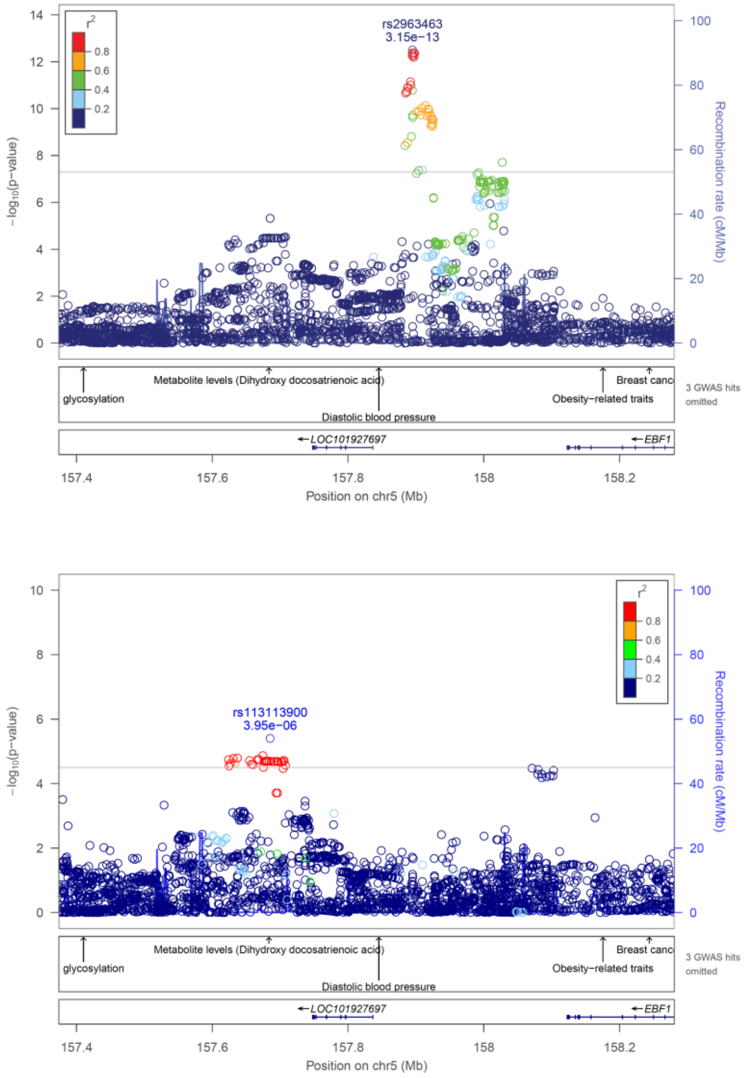

B. EEFSEC locus

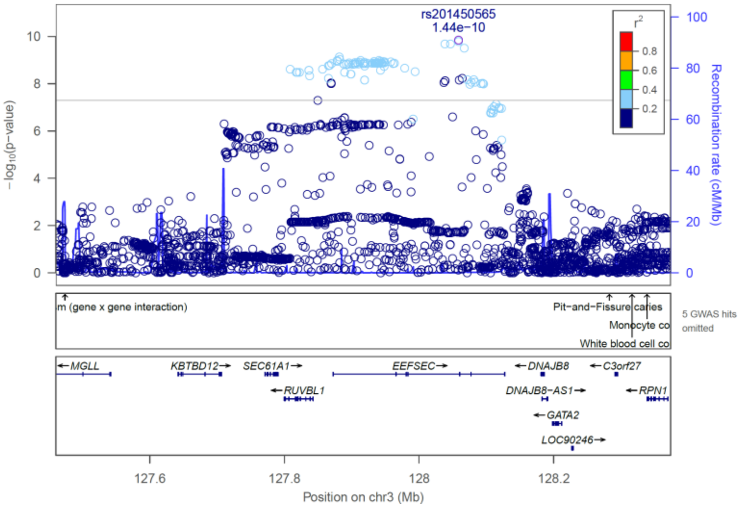

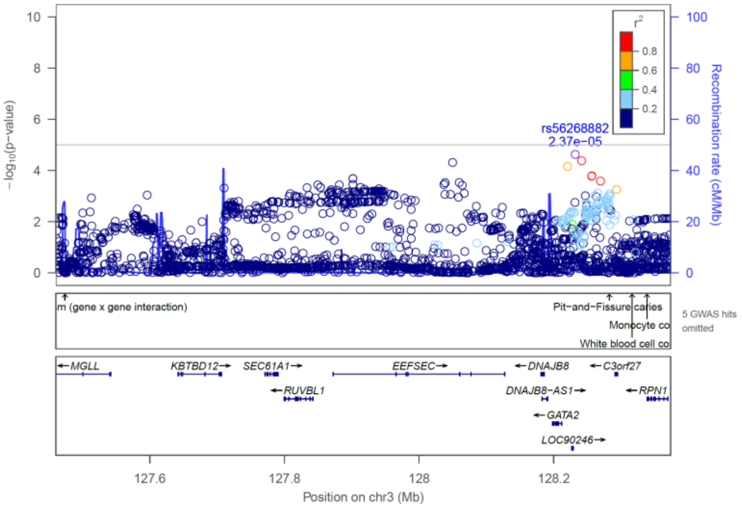

C. AGTR2 locus

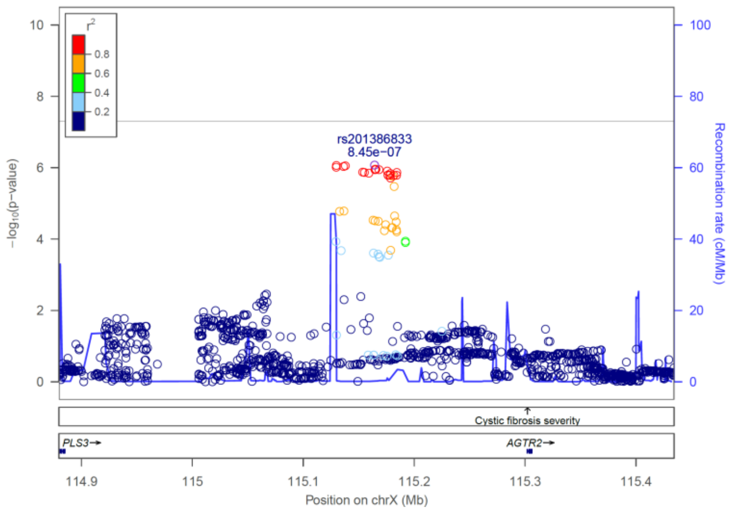

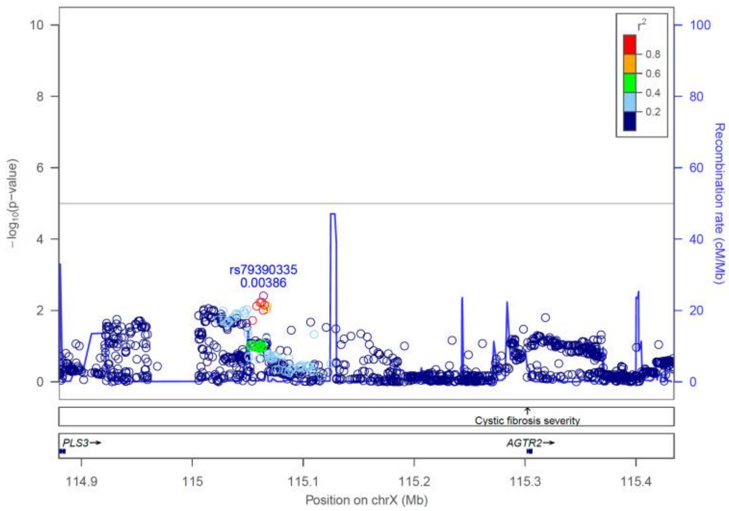

D. TEKT3 locus

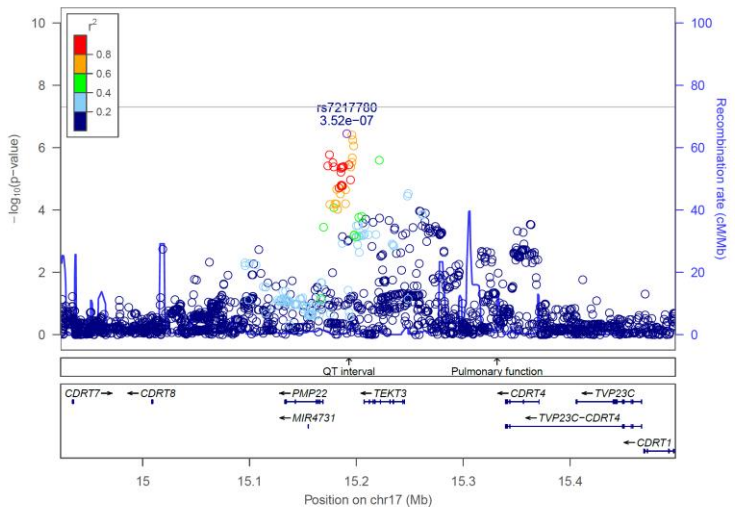

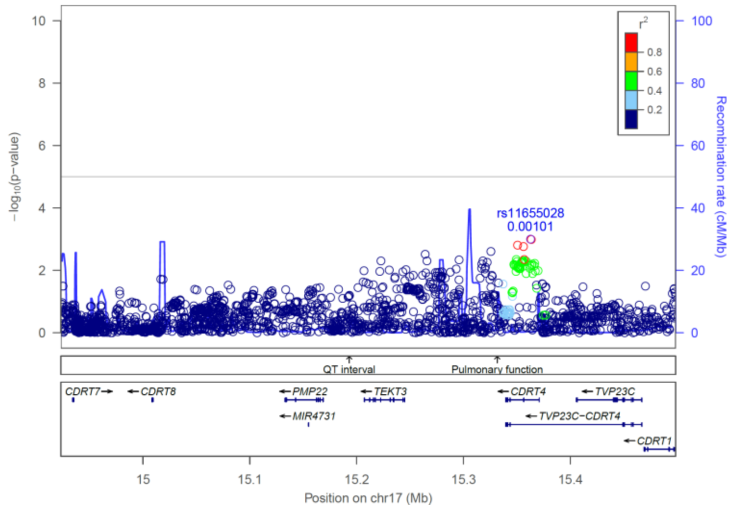

E. *TGFB1* locus

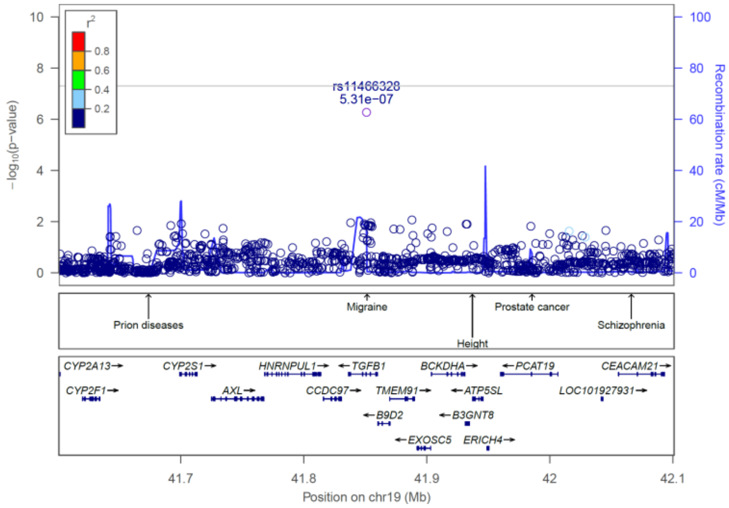

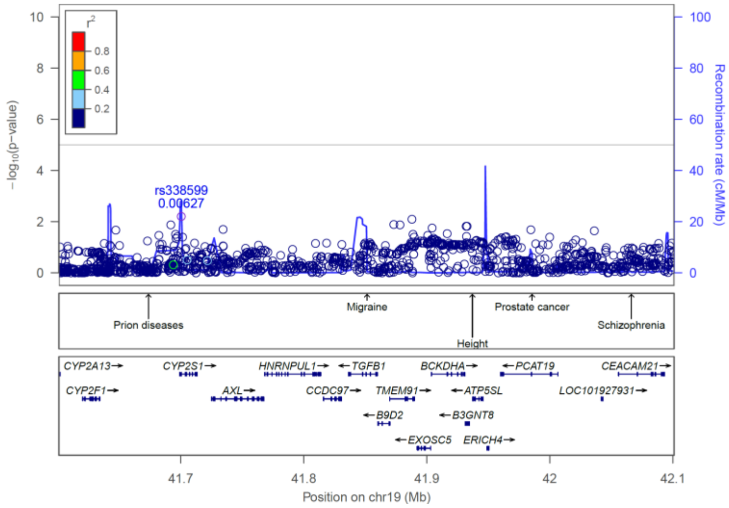

Figure S7. Variance explained in the replication studies using associated SNPs

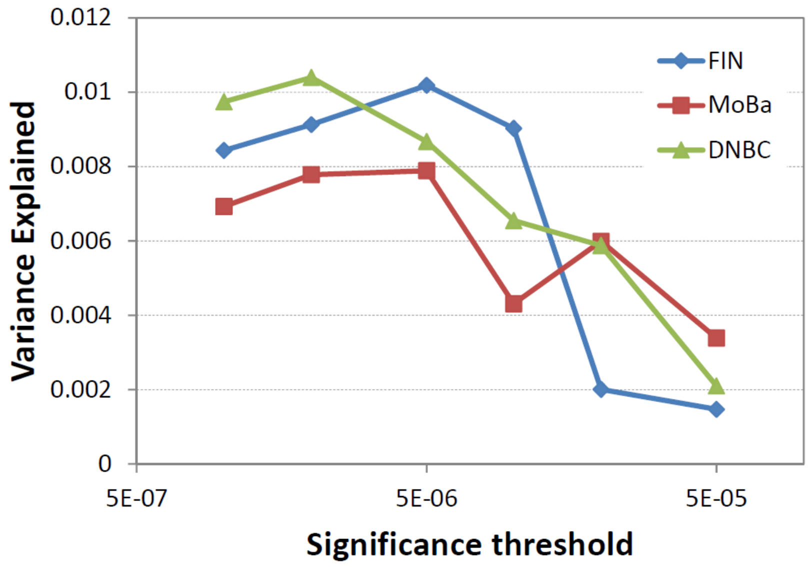

Figure S8. Manhattan plot of gene-wise association *P* values

A. Gestational age

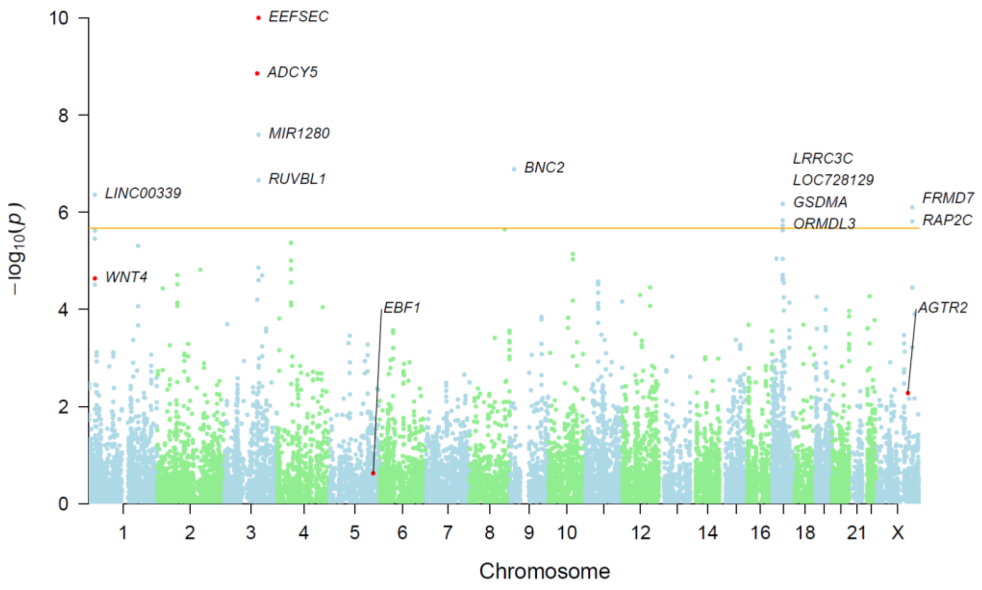

B. Preterm birth

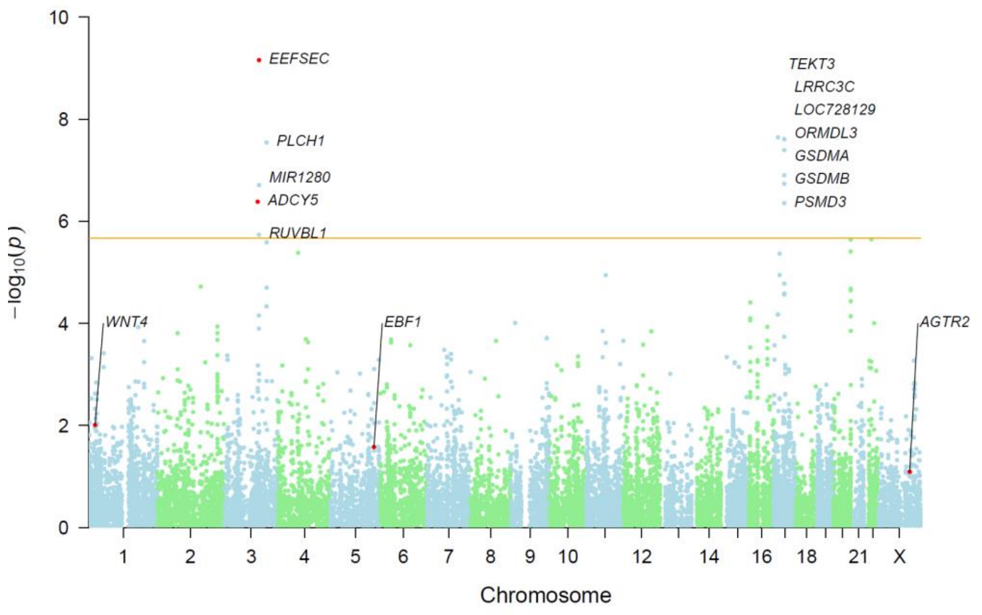

Figure S9. Gene set enrichment analysis showing enrichment of association signals in genes implicated in pregnancy related biological terms

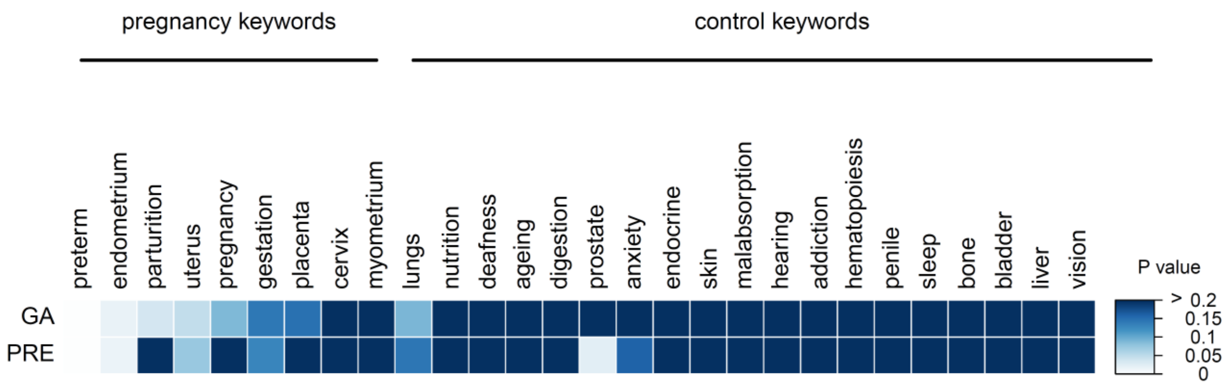

Enrichment of association signals (GA: gestational length; PRE: preterm birth) in gene sets related to 28 biological terms, including 9 pregnancy terms (left) and 19 "control" terms (right). For each biological term, we first constructed a set of genes related to that term by searching PubMed and then summarized the genomewide association SNP-level *P*-values in this set of genes into a gene-set-level *P* value (color-coded) using MAGMA.

Supplementary Tables

Table S1. Study datasets

Data set	Sample	Term	Preterm	Unclassified*	Total
Discovery data set					
23andMe@					
	Mother	40236	3331		43568
Replication studies					
FIN					
	Mother	554	334	0	888
	Infant	*564*	*253*	*0*	*817*
MoBa					
	Mother	928	906	0	1834
	Infant	*593*	*550*	*0*	*1143*
DNBC					
	Mother	4552	1325	44	5921
	Infant	*1015*	*1115*	*0*	*2130*
Total					
	Mother	6034	2565	44	8643
	Infant	*2172*	*1918*	*0*	*4090*

@ For preterm birth, our procedure attempted to maximize study power by preferentially retaining individuals with in case (preterm) group. For a continuous outcome (gestational age), our procedure attempted to keep as many unrelated people in the analysis as possible. This does mean that slightly different sets of women were included in the two analyses (the overlap is 99.8%). The total number shown here (N=43,568) was the number of discovery samples included in the analysis of gestational age.

* In the replication studies, individuals with gestational age between 36 and 37 weeks were categorized as “unclassified”. These individuals were included in the analysis of gestational age as a quantitative trait but excluded in the analysis of preterm birth as a dichotomous trait.

Table S2. Phenotype data of discovery samples (23andMe)

Phenotype	Age at pregnancy							
	(0,18]	(18,20]	(20,25]	(25,30]	(30,35]	(35,40]	(40,60]	Total
Gestational wks								
[22,37]*	318	522	1426	1493	1035	338	69	5201
(37,40]	1374	2472	7239	7243	4305	1437	281	24351
(40,41]	326	575	1532	1353	750	238	45	4819
(41,42]	406	820	2292	1940	964	301	40	6763
(42,45]	196	362	1017	590	215	43	11	2434
Total	2620	4751	13506	12619	7269	2357	446	43568
Preterm status								
Preterm	227	407	930	931	611	189	36	3331
Normal term	2387	4346	12581	11686	6655	2170	411	40236
Total	2614	4753	13511	12617	7266	2359	447	43567

* The number of individuals in the group with gestational wks [22, 37] (N=5,201) is larger than the number of individuals in the preterm group (N=3,331) because the former one includes 1,870 mothers with gestational wks = 37.

Table S3. Influences of co-variates in discovery samples

Gestational age

Covariate#	Estimate	s.e.	t value*	*P*-value
Age at pregnancy	−0.02860	0.00212	−13.5	2.3×10^{-41}
pc.0	−0.01834	0.01193	−1.5	0.12
pc.1	−0.01136	0.01188	−1.0	0.34
pc.2	−0.03916	0.01186	−3.3	0.00097
pc.3	−0.02635	0.01187	−2.2	0.026
pc.4	−0.02537	0.01185	−2.1	0.032
v2_platformv2	0.00647	0.06019	0.1	0.91
v3_1_platformv3.1	0.012	0.03257	0.4	0.71
v4_platformv4	−0.01168	0.03207	−0.4	0.72

Preterm birth

Covariate	Estimate	s.e.	LRT*	*P*-value
Age at pregnancy	0.00201	0.00323	0.4	0.53
pc.0	0.03238	0.01934	2.9	0.089
pc.1	−0.00910	0.0183	0.2	0.62
pc.2	0.03334	0.01814	3.3	0.068
pc.3	0.02746	0.01842	2.2	0.14
pc.4	0.02521	0.0187	1.9	0.17
v2_platformv2	−0.09468	0.09468	1	0.31
v3_1_platformv3.1	0.00369	0.0495	0	0.94
v4_platformv4	−0.01855	0.04887	0.1	0.7

# pc.0 – pc.4: first 5 components in PCA analysis. v2_platformv2, v3_1_platformv3.1 and v4_platformv4 are indicator variables for different genotyping platforms.

* Linear regression and Student's t-test was used to examine the influences of co-variates on gestational age as a quantitative trait. Logistic regression and likelihood ratio test (LRT) was used to examine the influences of co-variates on preterm birth as a dichotomous trait.

Table S4. Influence of covariates on gestational age (replication studies)

Covariate	FIN			MoBa			DNBC#			Meta*		
	**beta**	**se**	***P***	**beta**	**se**	***P***	**beta**	**se**	***P***	**beta**	**se**	***P***
Maternal age	0.183	0.176	0.296	0.287	0.138	0.037	-0.011	0.135	0.935	0.146	0.084	0.084
Infant gender	3.831	1.584	0.016	2.333	0.963	0.016	1.959	1.132	0.084	2.468	0.666	2.1E-4
Maternal height	0.333	0.144	0.021	0.476	0.086	2.99E-08	0.248	0.098	0.012	0.371	0.059	3.1E-10
Maternal weight	-0.113	0.073	0.124	-0.019	0.041	0.639	0.054	0.047	0.256	-0.007	0.029	0.811

Results from a subset of 1815 DNBC women corresponding to the original GENEVA preterm birth study are presented. In the rest DNBC samples some covariates were confounded due to the sample selection procedure.

*Meta shows the combined results from the three studies using fixed-effect meta-analysis.

Table S5. Suggestive GWA regions (discovery phase)

No	Chr	From	To	Length (kb)	Gene	Markers[@]	Association[#]				Functional[&]		GWAS[*]	
							Sig	+LD	Eff	*P*.cut[$]	All	LD	All	LD
Gestational age														
**1**	**5**	**157408195**	**158308569**	**900**	***EBF1***	**2897**	**119**	125	18	1.98E-04	1	0	11	0
**2**	**3**	**127460474**	**128372820**	**912**	***EEFSEC***	**2554**	**255**	279	33	1.08E-04	4	0	133	4
**3**	**X**	**114879442**	**115442159**	**563**	***AGTR2***	**1313**	**41**	41	9	3.97E-04	0	0	3	0
**4**	**1**	**22097396**	**22843805**	**746**	***WNT4***	**3015**	**46**	47	13	2.75E-04	29	0	16	3
**5**	**3**	**122668928**	**123372314**	**703**	***ADCY5***	**2091**	**7**	44	7	5.10E-04	3	0	10	0
6	2	73950833	74611290	660	*BOLA3*	1489	5	22	5	7.14E-04	7	0	4	0
7	9	16149953	16719213	569	*BNC2*	2134	6	13	6	5.95E-04	3	0	8	0
8	1	91984421	92490753	506	*TGFBR3*	1891	2	2	2	1.79E-03	6	0	6	0
9	9	101806013	102324119	518	*SEC61B*	1419	7	7	2	1.79E-03	3	0	6	0
10	6	30552017	31173629	622	*SFTA2*	5073	1	4	2	1.79E-03	141	0	64	0
**11**	**X**	**130986215**	**131826714**	**840**	***RAP2C***	**1285**	**1**	1	1	3.57E-03	3	0	0	0
12	10	28061621	28598815	537	*MPP7*	1870	1	8	2	1.79E-03	5	0	6	0
Preterm birth														
**1**	**5**	**157374012**	**158280813**	**907**	***EBF1***	**2983**	**102**	**114**	15	2.38E-04	0	0	11	0
**2**	**3**	**127460474**	**128373786**	**913**	***EEFSEC***	**2557**	**191**	**241**	25	1.43E-04	4	0	133	4
3	17	14923221	15498449	575	*TEKT3*	2043	4	16	6	5.95E-04	10	0	2	1
4	19	41601042	42101042	500	*TGFB1*	1457	1	1	1	3.57E-03	32	0	7	0
**5**	**X**	**114879714**	**115434372**	**555**	***AGTR2***	**1292**	**5**	**22**	5	7.14E-04	0	0	3	0

@: the total number of SNPs (MAF > 1%) in the region.

#: the number of association SNPs identified by discovery phase. **Sig**: number of SNPs with $P < 1\times10^{-6}$; **+LD**: number of SNPs with $P < 1\times10^{-6}$ and their close proxies ($r^2 > 0.8$); and **Eff**: the estimated "effective number of independent SNPs", which was used in Bonferroni correction for multiple testing.

$: *P*.cut: the Bonferroni corrected significance level for each region, calculated as 0.05 / Eff (effective number of independent SNPS) / number of regions (=14)

&: number of "functional" (including missense, nonsense, frame-shift and splicing) SNPs. **All**: all "functional" SNPs in the region; **LD**: number of "functional" SNPs which are in close LD ($r^2 > 0.8$) with the "Association" SNPs.

*: number of GWA SNPs by GWAS Catalog (V1.0.1, r2017-01-09). **All**: all GWA SNPs in the region; LD: number of GWA SNPs which are in close LD ($r^2 > 0.8$) with the "Association" SNPs.

Table S6. Association results obtained from "spontaneous only" and the full discovery data set.

Chr	Genes	SNP Information			Original[@]			Spontaneous only[#]		
		rs	pos	A/G	freq	eff	*P*	freq	eff	*P*
Gestational age										
**5**	***EBF1***	**rs2963463**	**157895049**	**C/T**	**0.272**	**-1.29**	**1.03E-21**	**0.272**	**-1.207**	**5.01E-28**
**3**	***EEFSEC***	**rs2955117**	**127881613**	**G/A**	**0.286**	**0.911**	**7.24E-12**	**0.286**	**0.604**	**2.33E-08**
**X**	***AGTR2***	**rs201226733**	**115164770**	**D/I**	**0.578**	**0.82**	**5.71E-11**	**0.579**	**0.701**	**5.63E-12**
**1**	***WNT4***	**rs56318008**	**22470407**	**C/T**	**0.139**	**1.05**	**1.15E-09**	**0.140**	**0.926**	**4.61E-11**
**3**	***ADCY5***	**rs4383453**	**123068359**	**G/A**	**0.2**	**-0.808**	**9.62E-08**	**0.199**	**-0.622**	**4.64E-07**
2	*BOLA3*	rs4853012	74361290	G/A	0.141	-0.92	1.05E-07	0.141	-0.655	3.44E-06
9	*BNC2*	rs717267	16408826	G/A	0.399	-0.637	1.67E-07	0.399	-0.463	3.08E-06
1	*TGFBR3*	rs4658267	92240753	C/A	0.319	0.679	1.87E-07	0.319	0.448	2.31E-05
9	*SEC61B*	rs182704	102068912	T/C	0.344	0.658	2.07E-07	0.345	0.564	4.35E-08
6	*SFTA2*	rs2532929	30897774	A/G	0.397	-0.622	4.19E-07	0.397	-0.386	1.12E-04
**X**	***RAP2C***	**rs200879388**	**131300571**	**D/I**	**0.649**	**0.662**	**4.52E-07**	**0.650**	**0.433**	**4.89E-05**
10	*MPP7*	rs2253165	28337017	A/G	0.44	-0.594	9.33E-07	0.440	-0.418	2.22E-05
Preterm birth										
**5**	***EBF1***	**rs2963463**	**157895049**	**C/T**	**0.272**	**1.23**	**3.15E-13**	**0.272**	**1.311**	**3.39E-14**
**3**	***EEFSEC***	**rs201450565**	**128058610**	**D/I**	**0.233**	**0.81**	**1.43E-10**	**0.233**	**0.831**	**7.10E-06**
17	*TEKT3*	rs7217780	15191024	T/C	0.336	1.15	3.52E-07	0.336	1.157	2.21E-05
19	*TGFB1*	rs11466328	41851042	G/A	0.0288	0.567	5.31E-07	0.0283	0.518	5.96E-06
**X**	***AGTR2***	**rs201386833**	**115164281**	**D/I**	**0.41**	**1.15**	**8.45E-07**	**0.408**	**1.175**	**3.59E-06**

@ Original (as reported in the manuscript): Women with a medical indication were excluded, but those who did not specify a medical indication were retained.

Spontaneous only: a subset of the original (full) data set, only including those who specifically checked "spontaneous birth".

Table S7. Discovery and replication of GWA loci associated with gestational age

No	Chr	From	To	Genes	SNP Information				Discovery Phase					Replication Phase				Genotypic			Joint analysis	
					rs	pos	A/B	AA	Freq	Eff	*P*-value	Rank	r^2	Freq	Eff	*P*-value	*P*.hetero	*P*.add	*P*.dom	*P*.geno	*P*-value	Directions
1	chr5	157408195	158308569	*EBF1*	**rs2963463**	**157895049**	**C/T**	**T**	**0.272**	**-1.29**	**1.03E-21**	**1**	-	**0.264**	**-1.11**	**0.00172**	**0.105**	**0.00308**	**0.36**	**0.00635**	**7.66E-24**	**+-+**
					rs201770678	157894745	D/I		0.267	-1.29	1.71E-21	2	0.865	0.275	-1.11	0.00184	0.117	0.245	0.314	0.477	1.37E-23	+-+
					rs12520982	157894747	T/C	T	0.266	-1.29	2.47E-21	3	0.848	0.281	-1.19	0.000844	0.151	0.0012	0.15	0.0043	8.72E-24	+++
					rs7729301[bwt]	157886953	A/G	G	0.265	-1.28	9.39E-21	13	0.915	0.256	-1.02	0.00426	0.0959	0.00802	0.396	0.0172	1.85E-22	+-+
					rs2946171	*157921940*	*T/G*	*T*	*0.219*	*-1.24*	*1.11E-17*	*24*	*0.708*	*0.206*	*-1.46*	*0.000138*	*0.0722*	*0.0156*	*0.822*	*0.000891*	*8.09E-21*	*+-+*
2	chr3	127460474	128372820	*EEFSEC RUVBL1 SEC61A1*	**rs2955117**	**127881613**	**G/A**	**A**	**0.286**	**0.911**	**7.24E-12**	**1**		**0.279**	**1.33**	**0.00016**	**0.895**	**1.23E-05**	**0.0411**	**5.26E-05**	**9.49E-15**	**+++**
					rs2999049	127878817	T/C	C	0.273	0.914	1.07E-11	2	0.955	0.27	1.42	5.51E-05	0.917	1.02E-05	0.0459	3.68E-05	6.68E-15	+++
					rs2461794	127870060	G/A	N	0.273	0.911	1.21E-11	3	0.861	0.276	1.4	7.71E-05	0.963	2.56E-05	0.103	6.75E-05	9.94E-15	+++
					rs200745338	*127869457*	*D/I*	-	*0.237*	*0.986*	*1.50E-11*	*8*	*0.724*	*0.232*	*1.91*	*7.62E-07*	*0.787*	*0.0236*	*0.236*	*0.0681*	*7.51E-16*	*+++*
					rs10934853[GWA]	128038373	C/A	A	0.274	0.894	2.43E-11	17	0.785	0.273	1.37	9.90E-05	0.837	2.05E-05	0.0583	7.22E-05	2.37E-14	+++
					rs2999052[GWA]	127892037	T/C	C	0.27	0.889	4.02E-11	24	0.949	0.269	1.44	4.36E-05	0.848	9.67E-06	0.0457	3.46E-05	2.39E-14	+++
					rs2687729[GWA]	127895226	A/G	G	0.27	0.889	4.04E-11	25	0.949	0.269	1.44	4.35E-05	0.849	9.74E-06	0.0452	3.51E-05	2.40E-14	+++
					rs2811474[GWA]	127892851	G/C	G	0.157	0.858	2.42E-07	234	0.424	0.16	1.45	0.000752	0.381	0.0153	0.597	0.00223	1.64E-09	+++
3	chrX	114879442	115442159	*AGTR2 PLS3*	**rs201226733**	**115164770**	**I/D**		**0.422**	**-0.82**	**5.71E-11**	**1**		**0.42**	**-1.67**	**9.18E-08**	**0.499**	**6.13E-05**	**0.208**	**0.000272**	**7.16E-16**	**+++**
					rs201386833	115164281	D/I		0.41	-0.837	6.52E-11	2	0.925	0.411	-1.7	9.24E-08	0.495	6.82E-05	0.192	0.00029	8.01E-16	+++
					rs35401609	115164771	I/D		0.421	-0.816	6.69E-11	3	1	0.42	-1.67	9.07E-08	0.5	6.13E-05	0.208	0.000272	8.45E-16	+++
					rs5950491	*115129714*	*C/A*	*C*	*0.423*	*-0.826*	*6.84E-11*	*5*	*0.917*	*0.425*	*-1.75*	*4.74E-08*	*0.479*	*5.76E-08*	*0.27*	*4.08E-07*	*6.62E-16*	*+++*
4	chr1	22097396	22843805	*WNT4 CDC42 CELA3A*	**rs56318008**	**22470407**	**C/T**	**C**	**0.139**	**1.05**	**1.15E-09**	**1**		**0.153**	**2.27**	**1.79E-07**	**0.162**	**0.0403**	**0.334**	**1.54E-06**	**3.35E-14**	**+++**
					rs55938609	22470451	G/C	G	0.139	1.05	1.15E-09	2	1	0.153	2.27	1.79E-07	0.162	0.0403	0.334	1.54E-06	3.32E-14	+++
					rs3820282[GWA]	22468215	C/T	C	0.144	1	4.25E-09	3	0.935	0.155	2.36	4.90E-08	0.171	0.0111	0.4	1.96E-07	8.15E-14	+++
					rs12037376	*22462111*	*G/A*	*g*	*0.145*	*1*	*4.45E-09*	*4*	*0.914*	*0.157*	*2.41*	*2.10E-08*	*0.136*	*0.00716*	*0.464*	*1.55E-07*	*5.55E-14*	*+++*
					rs3765350[GWA]	22447316	A/G	A	0.203	0.833	2.14E-08	8	0.633	0.21	1.88	6.41E-07	0.194	0.00316	0.414	6.65E-06	1.96E-12	+++
					rs2235529[GWA]	22450487	C/T	C	0.143	0.95	2.46E-08	10	0.896	0.159	2.31	6.32E-08	0.198	0.00505	0.715	1.14E-06	6.69E-13	+++
5	chr3	122668928	123372314	*ADCY5 SEC22A HACD2*	**rs4383453**	**123068359**	**G/A**	**G**	**0.2**	**-0.808**	**9.62E-08**	**1**		**0.197**	**-0.587**	**0.145**	**0.532**	**0.821**	**0.527**	**0.493**	**3.69E-08**	**-++**
					rs78519666	123065511	T/A	t	0.103	-1.14	2.64E-07	2	0.475	0.0986	-0.0876	0.881	0.281	0.0613	0.0906	0.174	1.14E-06	--+
					rs6794803	123085337	T/C	T	0.463	-0.6	5.52E-07	3	0.321	0.483	-1.12	0.000346	0.973	0.00046	0.406	0.00206	2.55E-09	+++
					rs9861425	*123072883*	*A/C*	*C*	*0.453*	*-0.598*	*6.12E-07*	*5*	*0.344*	*0.47*	*-1.38*	*9.52E-06*	*0.942*	*6.29E-06*	*0.0582*	*1.93E-05*	*4.24E-10*	*+++*
6	chr2	73950833	74611290	*BOLA3 MOB1A*	**rs4853012**	**74361290**	**G/A**	**G**	**0.141**	**-0.92**	**1.05E-07**	**1**		**0.145**	**-0.355**	**0.424**	**0.237**	**0.232**	**0.411**	**0.473**	**1.56E-07**	**-++**
					rs13390332	74207357	G/A	G	0.0579	-1.33	3.18E-07	2	0.129	0.0572	-2.05	0.00271	0.462	0.455	0.97	0.0297	5.09E-09	-++

				TET3	rs6721042	74217283	G/T	G	0.0587	-1.31	4.30E-07	3	0.129	0.0567	-2.06	0.00261	0.426	0.32	0.836	0.0278	6.90E-09	-++
					rs17009553	*74220035*	*G/A*	*G*	*0.0567*	*-1.28*	*1.14E-06*	*6*	*0.129*	*0.0565*	*-2.11*	*0.00204*	*0.43*	*0.319*	*0.88*	*0.0183*	*1.62E-08*	*-++*
7	chr9	16149953	16719213	*BNC2 C9orf92*	**rs717267**	**16408826**	**G/A**	**G**	**0.399**	**-0.637**	**1.67E-07**	**1**	-	**0.423**	**-0.12**	**0.703**	**0.498**	**0.853**	**0.52**	**0.763**	**5.25E-07**	**+-+**
					rs6475050	16407780	G/A	A	0.399	-0.63	2.44E-07	2	0.978	0.423	-0.096	0.76	0.483	0.947	0.46	0.737	8.46E-07	+-+
					rs10810563	16399953	G/T	T	0.408	-0.643	2.72E-07	3	0.882	0.425	-0.128	0.689	0.669	0.957	0.173	0.381	8.02E-07	+-+
					rs9298764	*16431230*	*A/G*	*G*	*0.456*	*-0.55*	*5.21E-06*	*19*	*0.807*	*0.474*	*-0.443*	*0.159*	*0.212*	*0.201*	*0.875*	*0.408*	*1.96E-06*	*+-+*
8	chr1	91984421	92490753	*TGFBR3 BRDT CDC7*	**rs4658267**	**92240753**	**C/A**	**C**	**0.319**	**0.679**	**1.87E-07**	**1**	-	**0.319**	**0.0562**	**0.867**	**0.706**	**0.993**	**0.603**	**0.84**	**8.76E-07**	**-++**
					rs4658265	*92240685*	*C/T*	*T*	*0.312*	*0.662*	*4.84E-07*	*2*	*0.914*	*0.311*	*0.125*	*0.71*	*0.94*	*0.974*	*0.454*	*0.696*	*1.43E-06*	*-++*
					rs284201	92240238	C/T	C	0.283	0.59	1.15E-05	3	0.802	0.269	-0.0151	0.966	0.998	0.535	0.32	0.61	4.36E-05	---
9	chr9	101806013	102324119	*SEC61B ALG2 TGFBR1*	***rs182704***	***102068912***	***T/C***	***C***	***0.344***	***0.658***	***2.07E-07***	***1***	-	***0.397***	***0.145***	***0.668***	***0.495***	***0.525***	***0.266***	***0.524***	***5.37E-07***	***--+***
					rs1622127	102063150	A/C	C	0.336	0.644	3.24E-07	2	0.764	0.351	0.0926	0.777	0.416	0.315	0.0935	0.236	1.11E-06	--+
					rs162621	102064575	T/C	C	0.336	0.643	3.53E-07	3	0.764	0.35	0.106	0.747	0.404	0.315	0.106	0.259	1.13E-06	--+
10	chr6	30552017	31173629	*SFTA2 VARS2 DPCR1*	**rs2532929**	**30897774**	**A/G**	**G**	**0.397**	**-0.622**	**4.19E-07**	**1**	-	**0.402**	**-0.0486**	**0.879**	**0.139**	**0.772**	**0.98**	**0.957**	**1.80E-06**	**+-+**
					rs2532927	30898434	A/G	G	0.367	-0.557	7.96E-06	2	0.902	0.377	-0.0603	0.85	0.413	0.773	0.827	0.952	2.34E-05	+-+
					rs2532926	*30898441*	*A/G*	*G*	*0.363*	*-0.556*	*9.02E-06*	*3*	*0.866*	*0.374*	*-0.0653*	*0.839*	*0.343*	*0.697*	*0.712*	*0.899*	*2.54E-05*	*+-+*
11	chrX	130986215	131826714	*RAP2C FRMD7 STK26*	***rs200879388***	***131300571***	***I/D***	-	***0.351***	***-0.662***	***4.52E-07***	***1***	-	***0.364***	***-1.1***	***0.000924***	***0.536***	***0.343***	***0.452***	***0.58***	***3.42E-09***	***+++***
					rs2999094	131268226	C/T	T	0.3	-0.596	5.59E-06	2	0.407	0.318	-1.27	0.00012	0.915	8.75E-05	0.182	0.0004	1.72E-08	+++
					rs2747027	131268092	T/G	T	0.3	-0.596	5.60E-06	3	0.407	0.318	-1.27	0.00012	0.916	8.75E-05	0.182	0.0004	1.72E-08	+++
12	chr10	28061621	28598815	*MPP7 ARMC4*	**rs2253165**	**28337017**	**A/G**	**G**	**0.44**	**-0.594**	**9.33E-07**	**1**	-	**0.429**	**0.454**	**0.154**	**0.456**	**0.258**	**0.855**	**0.527**	**4.50E-05**	**+--**
					rs2245244	*28316456*	*T/C*	*T*	*0.438*	*-0.561*	*3.69E-06*	*2*	*0.86*	*0.428*	*0.507*	*0.105*	*0.402*	*0.0948*	*0.743*	*0.248*	*0.00019*	*+--*
					rs2253011	28335832	C/T	C	0.434	-0.551	5.70E-06	3	0.885	0.423	0.486	0.125	0.369	0.101	0.532	0.243	0.000226	+--

For each region, we showed the top 3 (rank=1,2,3) SNPs based on their discovery phase *P* values. We also showed the SNP with the smallest replication *P*-value (*italic*), SNP with the smallest joint-analysis *P*-value (underlined) as well as previously reported GWA SNPs (marker by [GWA]) and birth weight associated SNPs (marker by [bwt]). *P*.hetero is *P* value of heterogeneity test across replication studies. *P*.add, *P*.dom and *P*.geno are *P* values for additive, dominant and genotypic effects based on genotype-based association test (d.f.=2). SNP positions were based on GRCh37/hg19. Alleles were given based on positive strand of reference genome. Allele B is used as the reference allele for frequency and effect.

Table S8. Discovery and replication of GWA loci associated with preterm birth

No	Chr	From	To	Genes	SNP Information				Discovery Phase					Replication Phase				Genotypic			Joint analysis	
					rs	pos	A/B	AA	Freq	Eff	*P*-value	Rank	r^2	Freq	Eff	*P*-value	*P*.hetero	*P*.add	*P*.dom	*P*.geno	*P*-value	Directions
1	chr5	157374012	158280813	*EBF1*	**rs2963463**	**157895049**	**C/T**	**T**	**0.272**	**1.23**	**3.15E-13**	**1**		**0.265**	**1.13**	**0.00153**	**0.103**	**0.0143**	**0.754**	**0.0503**	**4.53E-15**	**+-+**
					rs2964484	157897437	A/G	A	0.266	1.23	4.22E-13	2	0.934	0.265	1.14	0.000751	0.128	0.00252	0.626	0.00273	2.26E-15	+++
					rs2419911	157898103	G/T		0.266	1.23	4.22E-13	3	0.947	0.262	1.13	0.00133	0.124	0.00373	0.643	0.00396	5.72E-15	+-+
					rs12520982	157894747	T/C	T	0.266	1.23	5.52E-13	6	0.848	0.281	1.15	0.000562	0.0999	0.00323	0.56	0.00481	1.98E-15	+++
					rs7729301[bwt]	157886953	A/G	G	0.265	1.21	1.93E-11	14	0.915	0.257	1.12	0.00545	0.102	0.0226	0.771	0.0174	1.01E-12	+-+
					rs2946169	*157918959*	*C/T*	*T*	*0.217*	*1.22*	*1.09E-10*	*19*	*0.684*	*0.207*	*1.16*	*0.000551*	*0.0481*	*0.193*	*0.183*	*0.00179*	*2.20E-13*	*+-+*
2	chr3	127460474	128373786	*EEFSEC DNAJB8 GATA2*	**rs201450565**	**128058610**	**D/I**		**0.233**	**0.81**	**1.43E-10**	**1**		**0.135**	**0.824**	**0.00173**	**0.603**	**0.0603**	**0.279**	**0.0353**	**1.91E-12**	**+++**
					rs3849531	128058617	A/T	T	0.277	0.826	1.60E-10	2	0.414	0.267	0.852	9.95E-05	0.808	8.01E-05	0.0595	0.000182	1.48E-13	+++
					rs4857841	128046643	G/A	A	0.274	0.828	2.04E-10	3	0.383	0.272	0.846	2.79E-05	0.632	1.22E-05	0.0425	2.72E-05	5.30E-14	+++
					rs10934853[GWA]	128038373	C/A	A	0.274	0.828	2.04E-10	4	0.383	0.272	0.847	2.88E-05	0.632	1.31E-05	0.0441	2.81E-05	5.83E-14	+++
					rs2687729[GWA]	127895226	A/G	G	0.27	0.835	1.48E-09	61	0.28	0.269	0.836	7.84E-06	0.606	5.97E-06	0.0368	1.14E-05	9.10E-14	+++
					rs2999052[GWA]	127892037	T/C	C	0.27	0.835	1.49E-09	63	0.28	0.269	0.836	7.86E-06	0.606	5.82E-06	0.0384	1.07E-05	9.64E-14	+++
					rs200745338	*127869457*	*D/I*	-	*0.237*	*0.829*	*9.01E-09*	*95*	*0.239*	*0.232*	*0.797*	*3.52E-07*	*0.825*	*0.00618*	*0.406*	*0.00359*	*3.27E-14*	*+++*
					rs2811474[GWA]	127892851	G/C	G	0.157	0.84	2.70E-06	236	0.078	0.159	0.83	0.000165	0.377	0.00769	0.323	0.00101	2.80E-09	+++
3	chr17	14923221	15498449	*TEKT3 PMP22 TVP23C-CDRT4*	**rs7217780**	**15191024**	**T/C**	**C**	**0.336**	**1.15**	**3.52E-07**	**1**	-	**0.341**	**1.09**	**0.0252**	**0.488**	**0.103**	**0.55**	**0.106**	**4.85E-08**	**+++**
					rs66847224	15195812	D/I		0.344	1.15	3.98E-07	2	0.746	0.343	1.07	0.0647	0.208	0.203	0.791	0.211	2.10E-07	+++
					rs2024157	15196537	T/C	C	0.343	1.15	5.58E-07	3	0.752	0.343	1.07	0.0649	0.199	0.135	0.818	0.305	2.64E-07	+++
					rs1380181[GWA]	15193056	G/A	G	0.314	1.14	3.57E-06	10	0.908	0.327	1.08	0.0442	0.648	0.0927	0.986	0.144	7.68E-07	+++
					rs179521	*15173221*	*C/A*	*A*	*0.359*	*1.13*	*3.84E-06*	*11*	*0.805*	*0.357*	*1.1*	*0.0116*	*0.407*	*0.0114*	*0.798*	*0.0289*	*1.61E-07*	*+++*
4	chr19	41601042	42101042	*TGFB1 B9D2 TMEM91*	***rs11466328***	***41851042***	***G/A***	***G***	***0.0288***	***0.567***	***5.31E-07***	***1***	-	***0.0311***	***0.711***	***0.0377***	***0.685***	***NA***	***NA***	***NA***	***5.47E-07***	***+++***
					rs139581508	41888023	C/T	c	0.0146	1.31	0.00857	2	0.001	0.0208	0.92	0.532	0.361	NA	NA	NA	0.0703	++-
					rs8108632	41854534	A/T	t	0.458	1.07	0.011	3	0.012	0.447	0.924	0.0355	0.835	0.00744	0.314	0.0241	0.4	---
5	chrX	114879714	115434372	*AGTR2 PLS3*	**rs201386833**	**115164281**	**D/I**		**0.41**	**1.15**	**8.45E-07**	**1**		**0.41**	**1.18**	**2.31E-06**	**0.298**	**3.61E-05**	**0.497**	**0.000195**	**1.03E-11**	**+++**
					rs5991030	115129904	T/C	T	0.417	1.15	8.59E-07	2	0.851	0.422	1.18	1.72E-06	0.238	1.18E-06	0.271	5.67E-06	8.68E-12	+++
					rs62602480	115137917	C/T	C	0.419	1.14	8.77E-07	3	0.871	0.422	1.18	1.82E-06	0.249	1.32E-06	0.278	6.59E-06	9.27E-12	+++
					rs5950506	*115175748*	*G/A*	*G*	*0.42*	*1.14*	*1.22E-06*	*10*	*0.918*	*0.418*	*1.18*	*1.55E-06*	*0.304*	*9.02E-07*	*0.324*	*4.72E-06*	*1.12E-11*	*+++*

Notation: same as Table S6.

Table S9. Associations with known GWA SNPs

Locus	Chr	SNP	pos	A/B	freq	Trait	Effect	*P*	PMID
EBF1	5	rs7729301#	157886953	A/G	0.28	birth weight	-0.024	1.00E-8	27680694
EEFSEC	3	rs10934853	128038373	C/A	0.28	Prostate cancer	OR:1.12	3.00E-10	19767754
		rs2999052	127892037	T/C	0.26	Hypospadias	reduced OR:1.55	1.00E-26	25108383
		rs2687729	127895226	A/G	0.27	Menarche (age at onset)	0.04 unit increase	1.00E-10	25231870
					0.27	Menarche (age at onset)	2.3 week increase	1.00E-07	21102462
		rs2811474	127892851	G/C	NR	Schizophrenia	OR:1.064	9.00E-06	26198764
WNT4	1	rs3820282	22468215	C/T	NR	Epithelial ovarian cancer	OR:1.11	2.00E-08	25581431
		rs3765350	22447316	A/G	0.22	Bone mineral density	0.107 unit decrease	7.00E-10	24945404
		rs2235529	22450487	C/T	0.15	Bone mineral density	0.117 unit decrease	1.00E-08	24945404
					0.15	Endometriosis	OR:1.3	3.00E-09	23472165
		rs10917151*	22422721	G/A	0.15	Endometriosis	OR: 1.28	7.00E-09	23472165
		rs4654783*	22439520	C/T					
TEKT3	17	rs1380181	15193056	G/A	0.2	QT interval	1.97 unit decrease	3.00E-07	23166209

This table shows a list of SNPs which were significantly associated with gestational age or preterm birth and were also previously reported GWA SNPs (GWAS catalog, r2017-01-09). Effect and *P* are reported effect size and *P* value. Allele B was used as the reference allele for allele frequency and effect annotation. Except for rs77293021 (*EBF1* locus) and rs1380181 (*TEKT3* locus), the reference alleles (allele B) were associated with increased gestational age and reduced risk of preterm birth.

# This association was recently reported by Horikoshi et al. (*Nature* 2016, 538:248-52) and was not yet available in GWA catalog.

* rs10917151 and rs4654783 were listed in GWAS catalog not as individual records but as a 3-SNP haplotype together with rs2235529 and the frequency (0.15) is the reported haplotype frequency.

Table S10. eQTLs in GWA loci associated with gestational length

region#	Expression		eQTL SNP[@]						GWA SNP[$]				
	Gene	Tissue	rs	pos	beta	P_{QTL}	P_{Dis}	P_{Rep}	rs	pos	r^2	P_{Dis}	P_{Rep}
1	*THG1L*	Esophagus	rs1023666	157968677	-0.2	1.65E-05	4.41E-07	0.0684	rs3934712	157928196	0.83	9.91E-08	0.0434
2	*SEC61A1*	fibroblasts	rs6439111	127784612	-0.186	2.73E-13	8.99E-07	0.024	rs11717102	127727752	0.87	4.88E-07	0.0345
		Thyroid	rs1030655	127773676	-0.16	4.87E-07	8.95E-07	0.0296	rs11717102	127727752	0.95	4.88E-07	0.0345
	EEFSEC	Heart	rs7632756	127817508	0.374	2.96E-09	2.48E-11	4.19E-05	rs2955117	127881613	0.92	7.24E-12	0.0003
		Testis	rs4857868	127853473	0.52	1.37E-08	1.58E-08	0.00102	rs2999047	127878330	0.98	1.21E-08	0.00094
		Nerve	rs7355887	127808502	0.322	1.56E-08	2.25E-11	4.31E-05	rs2955117	127881613	0.89	7.24E-12	0.0003
		fibroblasts	rs7355887	127808502	0.264	6.93E-08	2.25E-11	4.31E-05	rs2955117	127881613	0.89	7.24E-12	0.0003
		Esophagus	rs2955117	127881613	-0.343	8.58E-08	7.24E-12	0.0003	rs2955117	127881613	1	7.24E-12	0.0003
		Whole_Blood	rs7632756	127817508	0.128	5.73E-07	2.48E-11	4.19E-05	rs2955117	127881613	0.92	7.24E-12	0.0003
		Adipose	rs2999049	127878817	-0.242	6.49E-07	1.07E-11	5.08E-05	rs2955117	127881613	0.96	7.24E-12	0.0003
		Lung	rs2811485	127919046	-0.243	1.51E-06	1.87E-10	5.39E-05	rs2955117	127881613	0.87	7.24E-12	0.0003
		Breast	rs2811485	127919046	-0.312	5.72E-06	1.87E-10	5.39E-05	rs2955117	127881613	0.87	7.24E-12	0.0003
		Skin	rs144609957	127869598	-0.172	1.33E-05	2.76E-11	0.0002	rs67451924	127867397	0.81	1.31E-11	4.25E-05
	RUVBL1	Testis	rs3849531	128058617	0.293	2.13E-08	2.71E-11	0.00035	rs4857841	128046643	0.87	2.42E-11	9.58E-05
		fibroblasts	rs1735546	128075398	0.095	1.26E-05	4.95E-09	6.39E-06	rs1735545	128081260	0.85	2.98E-09	4.42E-06
4	*LINC00339*	Testis	rs10917123	22355978	-0.574	5.47E-09	3.81E-08	1.23E-06	rs10917123	22355978	1	3.81E-08	1.23E-06
		Esophagus	rs10917123	22355978	-0.369	2.29E-06	3.81E-08	1.23E-06	rs10917123	22355978	1	3.81E-08	1.23E-06
		Skin	rs10917123	22355978	-0.345	7.41E-06	3.81E-08	1.23E-06	rs10917123	22355978	1	3.81E-08	1.23E-06
	WNT4	Thyroid	rs12037376	22462111	-0.389	3.16E-06	4.45E-09	2.33E-08	rs56318008	22470407	0.91	1.15E-09	2.32E-07
5	*SEC22A*	Esophagus	rs6771801	123082706	-0.541	2.66E-10	4.69E-06	0.0571	rs4383453	123068359	0.81	9.62E-08	0.164
		Adipose	rs11711950	123072318	-0.383	3.10E-09	1.01E-06	8.54E-05	rs6794803	123085337	0.9	5.52E-07	0.00023
		Artery	rs78519666	123065511	-0.747	3.38E-09	2.64E-07	0.794	rs78519666	123065511	1	2.64E-07	0.794
		Lung	rs78519666	123065511	-0.574	2.96E-08	2.64E-07	0.794	rs78519666	123065511	1	2.64E-07	0.794
		Nerve	rs6807089	123064089	-0.42	3.52E-08	1.54E-06	6.96E-05	rs9861425	123072883	0.85	6.12E-07	9.22E-06
		Thyroid	rs78519666	123065511	-0.535	9.21E-07	2.64E-07	0.794	rs78519666	123065511	1	2.64E-07	0.794
		Vagina	rs6771801	123082706	-0.626	1.45E-06	4.69E-06	0.0571	rs4383453	123068359	0.81	9.62E-08	0.164
		Testis	rs11711950	123072318	-0.37	2.82E-06	1.01E-06	8.54E-05	rs6794803	123085337	0.9	5.52E-07	0.00023

		Muscle	rs2062432	123078079	0.225	1.18E-05	1.30E-06	1.89E-05	rs6794803	123085337	0.89	5.52E-07	0.00023
	ADCY5	Artery	rs60414302	123095547	-0.27	3.03E-09	8.59E-06	0.0618	rs4383453	123068359	0.81	9.62E-08	0.164
		Heart	rs62265764	123094758	-0.335	3.18E-07	8.54E-06	0.0607	rs4383453	123068359	0.81	9.62E-08	0.164
		Lung	rs55942221	123092958	-0.263	5.98E-06	7.34E-06	0.0604	rs4383453	123068359	0.81	9.62E-08	0.164
	RP11-797D24.4	Artery	rs60414302	123095547	-0.251	4.52E-08	8.59E-06	0.0618	rs4383453	123068359	0.81	9.62E-08	0.164
		Heart	rs4677884	123062970	0.272	7.55E-08	1.60E-06	1.77E-05	rs9861425	123072883	0.88	6.12E-07	9.22E-06
6	*MGC10955**	Thyroid, Nerve, Adipose, …	rs2421752	74341615	1.44	1.04E-46	1.96E-06	0.869	rs4852329	74354544	0.89	9.18E-07	0.709
	*FNBP1P1**	Artery, Skin, Esophagus, …	rs2122290	74358901	0.815	7.10E-24	1.60E-06	0.242	rs4853012	74361290	0.81	1.05E-07	0.422
	DGUOK-AS1	Esophagus	rs6721042	74217283	-0.616	6.54E-08	4.30E-07	0.00256	rs13390332	74207357	1	3.18E-07	0.00277
		Skin	rs13390332	74207357	-0.475	6.89E-06	3.18E-07	0.00277	rs13390332	74207357	1	3.18E-07	0.00277
	INO80B	Artery	rs6721042	74217283	-0.449	5.58E-06	4.30E-07	0.00256	rs13390332	74207357	1	3.18E-07	0.00277
9	*KRT8P11*	Muscle	rs162621	102064575	-0.674	1.38E-25	3.53E-07	0.757	rs1622127	102063150	1	3.24E-07	0.79

This table shows the genes whose expression level were significantly associated (based on GTEx data) with the significant SNPs ($P < 1\times10^{-6}$, discovery stage) or their close proxies ($r^2 > 0.8$).

@ For each gene/tissue combination, only the most significant eQTL SNP (with smallest P_{QTL}) was shown. P_{QTL}: GTEx eQTL association P values; P_{Dis} and P_{Rep}: genetic association P values for the discovery and replication stage.

$ The most significant SNP (discovery stage) associate the phenotype that was in close LD ($r^2 > 0.8$) with the eQTL SNP.

* Expression levels of *MGC10955* (a hypothetical gene) and *FNBP1P1* (a pseudogene) were associated with the GWA SNPs in many tissues. Only the top one was shown to save space.

Table S11. eQTLs in GWA loci associated with preterm birth

Region#	Expression		eQTL SNP						GWA SNP				
	Gene	Tissue	rs	pos	beta	P_{QTL}	P_{Dis}	P_{Rep}	rs	pos	r^2	P_{Dis}	P_{Rep}
2	*EEFSEC*	Heart	rs7632756	127817508	0.374	2.96E-09	3.32E-09	9.57E-06	rs2955117	127881613	0.92	7.29E-10	8.46E-05
		Testis	rs4857868	127853473	0.52	1.37E-08	5.91E-07	0.00023	rs2811473	127884937	0.85	3.46E-07	8.66E-05
		Nerve	rs7355887	127808502	0.322	1.56E-08	2.39E-09	9.93E-06	rs2955117	127881613	0.89	7.29E-10	8.46E-05
		fibroblasts	rs7355887	127808502	0.264	6.93E-08	2.39E-09	9.93E-06	rs2955117	127881613	0.89	7.29E-10	8.46E-05
		Esophagus	rs2955117	127881613	-0.343	8.58E-08	7.29E-10	8.46E-05	rs2955117	127881613	1	7.29E-10	8.46E-05
		Whole_Blood	rs7632756	127817508	0.128	5.73E-07	3.32E-09	9.57E-06	rs2955117	127881613	0.92	7.29E-10	8.46E-05
		Adipose	rs2999049	127878817	-0.242	6.49E-07	1.15E-09	1.15E-05	rs4857841	128046643	0.83	2.04E-10	2.80E-05
		Lung	rs2811485	127919046	-0.243	1.51E-06	6.96E-09	2.06E-05	rs4857841	128046643	0.81	2.04E-10	2.80E-05
		Breast	rs2811485	127919046	-0.312	5.72E-06	6.96E-09	2.06E-05	rs4857841	128046643	0.81	2.04E-10	2.80E-05
		Skin	rs144609957	127869598	-0.172	1.33E-05	9.87E-09	3.11E-05	rs67451924	127867397	0.81	1.66E-09	9.38E-06
	RUVBL1	Testis	rs3849531	128058617	0.293	2.13E-08	1.60E-10	7.89E-05	rs3849531	128058617	1	1.60E-10	7.89E-05
		fibroblasts	rs1735546	128075398	0.095	1.26E-05	1.05E-08	7.75E-06	rs1735545	128081260	0.85	6.65E-09	1.14E-06
	SEC61A1	fibroblasts	rs4857837	127836319	-0.141	1.11E-06	2.71E-09	9.10E-06	rs2955117	127881613	0.92	7.29E-10	8.46E-05
3	*TEKT3*	Breast	rs2024157	15196537	-0.456	7.95E-07	5.58E-07	0.0646	rs66847224	15195812	0.99	3.98E-07	0.0638

Notation: similar to Table S10.

Table S12. Replication of GWA loci associated with gestational age using infant samples

No	Chr	From	To	Genes	SNP Information				Discovery Phase					Replication Phase				Joint analysis	
					rs	pos	A/B	AA	Freq	Eff	*P*-value	Rank	r^2	Freq	Eff	*P*-value	*P*.hetero	*P*-value	Directions
1	chr5	157408195	158308569	*EBF1*	**rs2963463**MC	**157895049**	**C/T**	**T**	**0.272**	**-1.29**	**1.03E-21**	**1**	-	**0.25**	**-0.372**	**0.503**	**0.294**	**2.87E-21**	**+-+**
					rs201770678	157894745	D/I		0.267	-1.29	1.71E-21	2	0.865	0.258	-0.317	0.57	0.439	5.94E-21	+-+
					rs12520982	157894747	T/C	T	0.266	-1.29	2.47E-21	3	0.848	0.264	-0.364	0.516	0.361	6.90E-21	+-+
					rs7729301bwt	157886953	A/G	G	0.265	-1.28	9.39E-21	13	0.915	0.241	-0.418	0.458	0.343	2.11E-20	+-+
					rs2946171MM	157921940	T/G	T	0.219	-1.24	1.11E-17	24	0.708	0.188	-0.891	0.146	0.546	4.37E-18	+++
					rs2964494	*157925562*	*A/G*		*0.223*	*-1.22*	*2.92E-17*	*33*	*0.695*	*0.191*	*-1.11*	*0.0687*	*0.391*	*5.45E-18*	*+++*
2	chr3	127460474	128372820	*EEFSEC RUVBL1 SEC61A1*	**rs2955117**	**127881613**	**G/A**	**A**	**0.286**	**0.911**	**7.24E-12**	**1**		**0.282**	**0.947**	**0.0724**	**0.692**	**1.39E-12**	**+++**
					rs2999049	127878817	T/C	C	0.273	0.914	1.07E-11	2	0.955	0.273	0.925	0.0795	0.629	2.20E-12	+++
					rs2461794	127870060	G/A	N	0.273	0.911	1.21E-11	3	0.861	0.278	0.85	0.111	0.649	3.35E-12	+++
					rs200745338MC	127869457	D/I	-	0.237	0.986	1.50E-11	8	0.724	0.233	1.36	0.0202	0.534	1.16E-12	+++
					rs10934853[GWA]	128038373	C/A	A	0.274	0.894	2.43E-11	17	0.785	0.276	0.868	0.0987	0.517	5.96E-12	+++
					rs2999052[GWA]	127892037	T/C	C	0.27	0.889	4.02E-11	24	0.949	0.272	0.986	0.0618	0.668	6.86E-12	+++
					rs2687729[GWA]	127895226	A/G	G	0.27	0.889	4.04E-11	25	0.949	0.272	0.985	0.0619	0.669	6.90E-12	+++
					rs2811474[GWA]	127892851	G/C	G	0.157	0.858	2.42E-07	234	0.424	0.157	1.73	0.00764	0.856	1.48E-08	+++
					rs72104653	*127971489*	*I/D*		*0.134*	*0.894*	*8.74E-07*	*248*	*0.445*	*0.15*	*2*	*0.00259*	*0.756*	*3.01E-08*	*+++*
3	chrX	114879442	115442159	*AGTR2 PLS3*	**rs201226733**	**115164770**	**I/D**	-	**0.422**	**-0.82**	**5.71E-11**	**1**	-	**0.428**	**-0.473**	**0.225**	**0.21**	**3.83E-11**	**++-**
					rs201386833	115164281	D/I		0.41	-0.837	6.52E-11	2	0.925	0.418	-0.431	0.279	0.145	5.79E-11	++-
					rs35401609	115164771	I/D		0.421	-0.816	6.69E-11	3	1	0.428	-0.472	0.226	0.208	4.45E-11	++-
					rs5950491MC	115129714	C/A	C	0.423	-0.826	6.84E-11	5	0.917	0.433	-0.5	0.211	0.221	4.19E-11	++-
					rs200165362	*115192158*	*I/D*		*0.487*	*0.667*	*1.81E-07*	*38*	*0.624*	*0.465*	*0.641*	*0.123*	*0.754*	*5.33E-08*	*+++*
4	chr1	22097396	22843805	*WNT4 CDC42 CELA3A*	***rs56318008***	***22470407***	***C/T***	***C***	***0.139***	***1.05***	***1.15E-09***	***1***		***0.155***	***1.55***	***0.0196***	***0.807***	***9.23E-11***	***+++***
					rs55938609MC	22470451	G/C	G	0.139	1.05	1.15E-09	2	1	0.155	1.55	0.0196	0.807	9.15E-11	+++
					rs3820282[GWA]	22468215	C/T	C	0.144	1	4.25E-09	3	0.935	0.157	1.34	0.0416	0.787	5.73E-10	+++
					rs12037376MM	22462111	G/A	g	0.145	1	4.45E-09	4	0.914	0.159	1.39	0.0334	0.799	5.18E-10	+++
					rs3765350[GWA]	22447316	A/G	A	0.203	0.833	2.14E-08	8	0.633	0.212	1.19	0.0396	0.375	2.93E-09	+++
					rs2235529[GWA]	22450487	C/T	C	0.143	0.95	2.46E-08	10	0.896	0.161	1.36	0.0365	0.863	3.16E-09	+++
5	chr3	122668928	123372314	*ADCY5 SEC22A HACD2*	**rs4383453**	**123068359**	**G/A**	**G**	**0.2**	**-0.808**	**9.62E-08**	**1**		**0.205**	**0.156**	**0.795**	**0.846**	**3.27E-07**	**+-+**
					rs78519666	123065511	T/A	t	0.103	-1.14	2.64E-07	2	0.475	0.103	0.752	0.379	0.63	1.91E-06	--+
					rs6794803	123085337	T/C	T	0.463	-0.6	5.52E-07	3	0.321	0.483	-0.735	0.12	0.154	1.64E-07	+++

					*rs9861425*MC	*123072883*	*A/C*	*C*	*0.453*	*-0.598*	*6.12E-07*	*5*	*0.344*	*0.468*	*-0.853*	*0.0713*	*0.107*	*1.31E-07*	*+-+*
6	chr2	73950833	74611290	*BOLA3 MOB1A TET3*	**rs4853012**	**74361290**	**G/A**	**G**	**0.141**	**-0.92**	**1.05E-07**	**1**	-	**0.145**	**-0.652**	**0.337**	**0.0064**	**7.02E-08**	**+-+**
					rs13390332MC	74207357	G/A	G	0.0579	-1.33	3.18E-07	2	0.129	0.0554	-0.854	0.428	0.548	2.51E-07	+-+
					rs6721042	74217283	G/T	G	0.0587	-1.31	4.30E-07	3	0.129	0.0549	-1.12	0.3	0.489	2.50E-07	+-+
					rs17009553MM	74220035	G/A	G	0.0567	-1.28	1.14E-06	6	0.129	0.0548	-1.14	0.29	0.483	6.42E-07	+-+
					rs71418714	*74219886*	*G/C*	*G*	*0.0549*	*-1.27*	*2.19E-06*	*24*	*0.152*	*0.0537*	*-1.25*	*0.248*	*0.419*	*1.09E-06*	*+-+*
7	chr9	16149953	16719213	*BNC2 C9orf92*	**rs717267**MC	**16408826**	**G/A**	**G**	**0.399**	**-0.637**	**1.67E-07**	**1**		**0.415**	**-0.433**	**0.37**	**0.601**	**1.20E-07**	**++-**
					rs6475050	16407780	G/A	A	0.399	-0.63	2.44E-07	2	0.978	0.415	-0.31	0.523	0.653	2.43E-07	++-
					rs10810563	16399953	G/T	T	0.408	-0.643	2.72E-07	3	0.882	0.41	-0.658	0.186	0.642	1.11E-07	+++
					*rs9298764*MM	*16431230*	*A/G*	*G*	*0.456*	*-0.55*	*5.21E-06*	*19*	*0.807*	*0.462*	*-0.707*	*0.145*	*0.794*	*1.82E-06*	+++
8	chr1	91984421	92490753	*TGFBR3 BRDT CDC7*	**rs4658267**MC	**92240753**	**C/A**	**C**	**0.319**	**0.679**	**1.87E-07**	**1**	-	**0.329**	**-0.0183**	**0.972**	**0.221**	**4.64E-07**	**--+**
					*rs4658265*MM	*92240685*	*C/T*	*T*	*0.312*	*0.662*	*4.84E-07*	*2*	*0.914*	*0.322*	*0.147*	*0.774*	*0.344*	*7.57E-07*	*+-+*
					rs284201	92240238	C/T	C	0.283	0.59	1.15E-05	3	0.802	0.283	-0.0398	0.941	0.365	2.27E-05	--+
9	chr9	101806013	102324119	*SEC61B ALG2 TGFBR1*	***rs182704***MC	***102068912***	***T/C***	***C***	***0.344***	***0.658***	***2.07E-07***	***1***	-	***0.398***	***0.414***	***0.42***	***0.14***	***1.65E-07***	***-++***
					rs1622127	102063150	A/C	C	0.336	0.644	3.24E-07	2	0.764	0.35	0.317	0.521	0.393	3.23E-07	-++
					rs162621	102064575	T/C	C	0.336	0.643	3.53E-07	3	0.764	0.35	0.329	0.506	0.386	3.44E-07	-++
10	chr6	30552017	31173629	*SFTA2 VARS2 DPCR1*	**rs2532929**MC	**30897774**	**A/G**	**G**	**0.397**	**-0.622**	**4.19E-07**	**1**	-	**0.413**	**-0.08**	**0.87**	**0.378**	**7.56E-07**	**++-**
					rs2532927	*30898434*	*A/G*	*G*	*0.367*	*-0.557*	*7.96E-06*	*2*	*0.902*	*0.387*	*0.101*	*0.837*	*0.752*	*1.89E-05*	*++-*
					rs2532926MM	30898441	A/G	G	0.363	-0.556	9.02E-06	3	0.866	0.383	0.0937	0.849	0.763	2.08E-05	++-
11	chrX	130986215	131826714	*RAP2C FRMD7 STK26*	***rs200879388***MC	***131300571***	***I/D***	-	***0.351***	***-0.662***	***4.52E-07***	***1***	-	***0.357***	***-0.727***	***0.0859***	***0.969***	***9.87E-08***	***+++***
					rs2999094	131268226	C/T	T	0.3	-0.596	5.59E-06	2	0.407	0.312	-0.813	0.0527	0.617	8.98E-07	+++
					rs2747027	131268092	T/G	T	0.3	-0.596	5.60E-06	3	0.407	0.312	-0.814	0.0524	0.617	8.97E-07	+++
12	chr10	28061621	28598815	*MPP7 ARMC4*	**rs2253165**MC	**28337017**	**A/G**	**G**	**0.44**	**-0.594**	**9.33E-07**	**1**	-	**0.427**	**0.522**	**0.29**	**0.891**	**6.43E-06**	**---**
					rs2245244MM	28316456	T/C	T	0.438	-0.561	3.69E-06	2	0.86	0.421	0.471	0.333	0.673	2.07E-05	+--
					rs2253011	*28335832*	*C/T*	*C*	*0.434*	*-0.551*	*5.70E-06*	*3*	*0.885*	*0.416*	*0.596*	*0.228*	*0.802*	*3.81E-05*	*---*

Notation: similar to Table S7. For each locus, the SNP with the smallest replication *P*-value in infants is shown in *italic*; the index SNP (the SNP with the smallest discovery *P*-value in 23andMe mothers) is shown in **bold**, and the SNP with the smallest joint-analysis *P*-value is underlined. SNPs with smallest joint *P*-value and replication *P*-value in maternal association test (Table S6) are labeled by MC and MM, respectively.

Table S13. Replication of GWA loci associated with preterm birth using infant samples

No	Chr	From	To	Genes	SNP Information				Discovery Phase					Replication Phase				Joint analysis	
					rs	pos	A/B	AA	Freq	Eff	*P*-value	Rank	r^2	Freq	Eff	*P*-value	*P*.hetero	*P*-value	Directions
1	chr5	157374012	158280813	*EBF1*	**rs2963463**	**157895049**	**C/T**	**T**	**0.272**	**1.23**	**3.15E-13**	**1**		**0.25**	**1.04**	**0.402**	**0.416**	**5.30E-12**	**+-+**
					rs2964484	157897437	A/G	A	0.266	1.23	4.22E-13	2	0.934	0.248	1.06	0.289	0.458	3.28E-12	+-+
					rs2419911	157898103	G/T		0.266	1.23	4.22E-13	3	0.947	0.247	1.05	0.377	0.441	7.26E-12	+-+
					rs12520982MC	157894747	T/C	T	0.266	1.23	5.52E-13	6	0.848	0.264	1.04	0.405	0.444	8.60E-12	+-+
					rs7729301bwt	157886953	A/G	G	0.265	1.21	1.93E-11	14	0.915	0.241	1.06	0.302	0.34	1.02E-10	+-+
					rs2946169MM	157918959	C/T	T	0.217	1.22	1.09E-10	19	0.684	0.189	1.04	0.446	0.907	7.50E-10	+++
					rs11135035	*157900829*	*A/G*	*G*	*0.358*	*1.16*	*5.79E-08*	*46*	*0.393*	*0.333*	*1.06*	*0.195*	*0.388*	*7.16E-08*	*+-+*
2	chr3	127460474	128373786	*EEFSEC DNAJB8 GATA2*	**rs201450565**	**128058610**	**D/I**		**0.233**	**0.81**	**1.43E-10**	**1**		**0.135**	**1**	**0.972**	**0.316**	**7.75E-09**	**-+-**
					rs3849531	128058617	A/T	T	0.277	0.826	1.60E-10	2	0.414	0.27	0.95	0.314	0.524	2.75E-09	++-
					rs4857841	128046643	G/A	A	0.274	0.828	2.04E-10	3	0.383	0.276	0.936	0.191	0.35	1.55E-09	++-
					rs10934853GWA	128038373	C/A	A	0.274	0.828	2.04E-10	4	0.383	0.276	0.938	0.198	0.337	1.75E-09	++-
					rs2687729GWA	127895226	A/G	G	0.27	0.835	1.48E-09	61	0.28	0.272	0.929	0.144	0.488	4.58E-09	+++
					rs2999052GWA	127892037	T/C	C	0.27	0.835	1.49E-09	63	0.28	0.272	0.929	0.143	0.488	4.77E-09	+++
					rs200745338MC	127869457	D/I		0.237	0.829	9.01E-09	95	0.239	0.233	0.916	0.113	0.569	1.36E-08	+++
					rs2248668	*127890749*	*A/G*	*G*	*0.151*	*0.831*	*9.71E-07*	*190*	*0.091*	*0.145*	*0.861*	*0.0191*	*0.897*	*1.01E-07*	*+++*
					rs2811474GWA	127892851	G/C	G	0.157	0.84	2.70E-06	236	0.078	0.157	0.885	0.0488	0.594	7.23E-07	+++
3	chr17	14923221	15498449	*TEKT3 PMP22 TVP23C-CDRT4*	**rs7217780**MC	**15191024**	**T/C**	**C**	**0.336**	**1.15**	**3.52E-07**	**1**	-	**0.33**	**1.08**	**0.112**	**0.884**	**1.57E-07**	**+++**
					rs66847224	15195812	D/I		0.344	1.15	3.98E-07	2	0.746	0.335	1.06	0.192	0.669	4.00E-07	+++
					rs2024157	15196537	T/C	C	0.343	1.15	5.58E-07	3	0.752	0.334	1.06	0.183	0.672	4.87E-07	+++
					rs1380181GWA	15193056	G/A	G	0.314	1.14	3.57E-06	10	0.908	0.318	1.08	0.0994	0.794	1.13E-06	+++
					rs179521MM	15173221	C/A	A	0.359	1.13	3.84E-06	11	0.805	0.352	1.08	0.109	0.858	1.36E-06	+++
					rs9944503	*15185523*	*G/A*	*G*	*0.326*	*1.14*	*4.56E-06*	*18*	*0.83*	*0.329*	*1.09*	*0.0735*	*0.719*	*1.13E-06*	*+++*
4	chr19	41601042	42101042	*TGFB1 B9D2 TMEM91*	***rs11466328***MC	***41851042***	***G/A***	***G***	***0.0288***	***0.567***	***5.31E-07***	***1***	-	***0.0303***	***0.879***	***0.539***	***0.381***	***1.22E-05***	***++-***
					rs139581508	41888023	C/T	c	0.0146	1.31	0.00857	2	0.001	0.0197	0.866	0.397	0.598	0.0539	+--
					rs8108632	41854534	A/T	t	0.458	1.07	0.011	3	0.012	0.443	1.09	0.0649	0.865	0.0019	+++
5	chrX	114879714	115434372	*AGTR2 PLS3*	**rs201386833**	**115164281**	**D/I**		**0.41**	**1.15**	**8.45E-07**	**1**		**0.418**	**1.02**	**0.524**	**0.0764**	**1.33E-05**	**++-**
					rs5991030MC	115129904	T/C	T	0.417	1.15	8.59E-07	2	0.851	0.43	1.03	0.433	0.108	8.83E-06	++-
					rs62602480	115137917	C/T	C	0.419	1.14	8.77E-07	3	0.871	0.431	1.03	0.449	0.0992	1.03E-05	++-
					rs5950506MM	115175748	G/A	G	0.42	1.14	1.22E-06	10	0.918	0.427	1.03	0.416	0.0881	1.06E-05	++-
					rs6608550	*115178556*	*A/G*		*0.422*	*1.14*	*1.46E-06*	*15*	*0.912*	*0.43*	*1.03*	*0.405*	*0.085*	*1.19E-05*	*++-*

Notation: similar to Table S12.

Table S14. Comparison of effect sizes estimated from mothers and infants

Region	Chr	SNP	pos	alleles	Mother			Infant		
					eff	se	beta	eff	se	beta
Gestational age										
EBF1	5	rs2946171	157921940	T/G	-1.46	0.38	-3.81	-0.89	0.61	-1.45
EEFSEC	3	rs200745338	127869457	D/I	1.91	0.39	4.94	1.36	0.59	2.32
AGTR2	X	rs5950491	115129714	C/A	-1.75	0.32	-5.47	-0.50	0.40	-1.25
WNT4	1	rs12037376	22462111	G/A	2.41	0.43	5.60	1.39	0.65	2.13
ADCY5	3	rs9861425	123072883	A/C	-1.38	0.31	-4.42	-0.85	0.47	-1.80
RAP2C	X	rs200879388	131300571	I/D	-1.10	0.33	-3.31	-0.73	0.42	-1.72
BOLA3	2	rs17009553	74220035	G/A	-2.11	0.68	-3.08	-1.14	1.08	-1.06
*Preterm birth**										
EBF1	5	rs2946169	157918959	C/T	-0.15	0.04	-3.50	-0.04	0.06	-0.68
EEFSEC	3	rs200745338	127869457	D/I	0.23	0.04	5.09	0.10	0.05	1.93
AGTR2	X	rs5950506	115175748	G/A	-0.17	0.03	-4.76	-0.03	0.04	-0.81
TEKT3	17	rs179521	15173221	C/A	-0.10	0.04	-2.60	-0.08	0.05	-1.63
TGFB1	19	rs11466328	41851042	G/A	0.37	0.14	2.68	0.12	0.17	0.72

* For gestational age, effect is unstandardized regression coefficient, which shows the estimated changes in gestational days per allele. The effect size for preterm birth was given by log(OR). Beta is the standardized coefficient.

Table S15. Joint maternal and fetal genetic association analysis in mother/infant pairs

Locus	chr	rs	pos	alleles	Maternal*				Fetal			
					eff	se	beta	*P*	eff	se	beta	*P*
Gestational age												
EBF1	5	rs2946171	157921940	T/G	-0.880	0.722	-1.218	0.223	0.121	0.761	0.159	0.874
EEFSEC	3	rs200745338	127869457	D/I	1.060	0.689	1.538	0.124	0.725	0.679	1.068	0.286
AGTR2	X	rs5950491	115129714	C/A	-1.995	0.650	-3.070	**0.002**	0.129	0.525	0.246	0.806
WNT4	1	rs12037376	22462111	G/A	2.885	0.793	3.638	**0.0003**	0.168	0.786	0.214	0.831
ADCY5	3	rs9861425	123072883	A/C	-0.979	0.594	-1.649	0.099	-0.545	0.582	-0.936	0.349
RAP2C	X	rs200879388	131300571	I/D	-1.026	0.665	-1.543	0.123	-0.038	0.550	-0.068	0.945
BOLA3	2	rs17009553	74220035	G/A	-2.526	1.340	-1.885	0.059	-0.034	1.361	-0.025	0.980
Preterm birth												
EBF1	5	rs2946169	157918959	C/T	0.127	0.069	1.839	0.066	-0.048	0.073	-0.652	0.514
EEFSEC	3	rs200745338	127869457	D/I	-0.169	0.068	-2.496	**0.013**	-0.026	0.066	-0.397	0.692
AGTR2	X	rs5950506	115175748	G/A	0.188	0.063	2.974	**0.003**	-0.036	0.051	-0.699	0.484
TEKT3	17	rs179521	15173221	C/A	0.117	0.059	1.979	**0.048**	0.053	0.059	0.904	0.366
TGFB1	19	rs11466328	41851042	G/A	-0.507	0.453	-1.119	0.263	0.061	0.316	0.193	0.847

* Because the number of mother/infant pairs (N=3,184) used in this joint maternal/fetal genetic association analysis is much smaller than the number of mothers (N=8,643) used in the maternal only analysis, some of the loci were not significant for the maternal association. However, for the significant or marginal significant ones (highlighted in bold), the associations were exclusively observed in mothers supporting the maternal contribution.

Table S16. Conditional association tests of replicated loci

Region	Chr	Primary			Secondary					
		SNP	Pos	*P*	SNP	Pos	*P*	Dist (kb)	$r^{2@}$	$\|D'\|^{\#}$
Gestational age										
EBF1	5	rs2963463	157895049	1.03E-21	rs13172197	157736918	2.40E-06	158.1	0.005	0.144
EEFSEC	3	rs2955117	127881613	7.24E-12	rs7431308	128049793	4.53E-05	168.2	0.051	0.523
AGTR2	X	rs201226733	115164770	5.71E-11	rs12556792	115129442	0.00373	35.3	0.237	1.000
WNT4	1	rs56318008	22470407	1.15E-09	rs140353641	22593805	0.000191	123.4	0.053	0.530
ADCY5	3	rs4383453	123068359	9.62E-08	rs78661226	122918928	4.71E-05	149.4	0.050	0.743
RAP2C	X	rs200879388	131300571	4.52E-07	rs191919091	131530470	0.00289	229.9	0.010	1.000
BOLA3	2	rs4853012	74361290	1.05E-07	rs10166357	74230656	9.21E-05	130.6	0.040	0.209
Preterm birth										
EBF1	5	rs2963463	157895049	3.15E-13	rs113113900	157685341	3.95E-06	209.7	0.001	0.087
EEFSEC	3	rs201450565	128058610	1.44E-10	rs56268882	128231953	2.37E-05	173.3	0.019	0.185
AGTR2	X	rs201386833	115164281	8.45E-07	rs79390335	115064123	0.00386	100.2	0.000	0.025
TEKT3	17	rs7217780	15191024	3.52E-07	rs11655028	15362705	0.00101	171.7	0.017	0.751
TGFB1	19	rs11466328	41851042	5.31E-07	rs338599	41700493	0.00627	150.5	0.000	0.034

* The secondary SNPs were identified by GCTA-COJO --cojo-cond analysis conditioning on the primary SNP (index SNP with the smallest *P* value).

@ The r^2 was estimated based on genotypic allele counts of the reference 1000 Genome Phase 1 EUR samples.

The Lewontin's |*D'*| was calculated based on haplotype frequencies estimated via the EM algorithm.

Table S17. Variance explained by all common SNPs (MAF > 0.01) estimated using GCTA

	Gestational age					Preterm birth				
	Vg/Vp	SE	LRT	*P*	n	Vg/Vp_L	SE	LRT	*P*	n
FIN	0.379	0.281	2.234	0.067	888	0.681	0.324	5.135	0.012	888
MoBa	0.121	0.153	0.766	0.191	1834	0.153	0.178	0.784	0.188	1834
DNBC	0.165	0.057	8.908	0.001	5921	0.211	0.087	6.291	0.006	5756
Meta	0.167	0.052	10.163	0.001		0.226	0.076	8.921	0.001	

* The estimate of variance explained was transformed to the underlying liability scale.

# The meta results were calculated using fixed effect inverse variance approach.

Table S18. Variance explained in the replication studies using GWA SNPs identified from discovery data set (23andMe)

α	Markers*	Variance explained (%)			
		FIN	MoBa	DNBC	Avg
1.0E-06	12	0.84%	0.69%	0.97%	0.84%
2.0E-06	13	0.91%	0.78%	1.04%	0.91%
5.0E-06	18	1.02%	0.79%	0.87%	0.87%
1.0E-05	29	0.90%	0.43%	0.65%	0.61%
2.0E-05	41	0.20%	0.60%	0.59%	0.52%
5.0E-05	114	0.15%	0.34%	0.21%	0.25%

*The number GWA SNPs identified at different levels of significance (α) from discovery (23andMe) data set.

Table S19. Difference in gestational length between individuals with high (top quartile) and low (bottom quartile) genetic scores

	Bottom quartile*		Top quartile		Difference		
	**mean**	**sd**	**mean**	**sd**	**mean**	**95% CI**	***P*-value**
FIN	262.12	23.44	267.41	23.17	5.29	(0.94,9.64)	0.017
MoBa	261.19	20.28	264.64	20.32	3.45	(0.82,6.08)	0.01012
DNBC	257.40	27.51	264.36	25.03	6.96	(3.61,10.31)	0.00005
Meta	259.81	24.14	265.06	22.91	5.24	(3.32,7.16)	9.39E-08

* The top and bottom genetic quartiles were selected based on a genetic score calculated using the 12 index SNPs with discovery *P*-value < 1E-6

Supplementary References

1. Durand EY, Do CB, Mountain JL, Macpherson JM. Ancestry Composition: A Novel, Efficient Pipeline for Ancestry Deconvolution. bioRxiv 2014.

2. Henn BM, Hon L, Macpherson JM, et al. Cryptic distant relatives are common in both isolated and cosmopolitan genetic samples. PLoS One 2012;7:e34267.

3. Browning SR, Browning BL. Rapid and accurate haplotype phasing and missing-data inference for whole-genome association studies by use of localized haplotype clustering. American journal of human genetics 2007;81:1084-97.

4. Fuchsberger C, Abecasis GR, Hinds DA. minimac2: faster genotype imputation. Bioinformatics 2015;31:782-4.

5. Plunkett J, Doniger S, Orabona G, et al. An evolutionary genomic approach to identify genes involved in human birth timing. PLoS genetics 2011;7:e1001365.

6. Magnus P, Birke C, Vejrup K, et al. Cohort Profile Update: The Norwegian Mother and Child Cohort Study (MoBa). International journal of epidemiology 2016;45:382-8.

7. Myking S, Boyd HA, Myhre R, et al. X-chromosomal maternal and fetal SNPs and the risk of spontaneous preterm delivery in a Danish/Norwegian genome-wide association study. PLoS One 2013;8:e61781.

8. Olsen J, Melbye M, Olsen SF, et al. The Danish National Birth Cohort--its background, structure and aim. Scandinavian journal of public health 2001;29:300-7.

9. Ryckman KK, Feenstra B, Shaffer JR, et al. Replication of a genome-wide association study of birth weight in preterm neonates. The Journal of pediatrics 2012;160:19-24 e4.

10. Nohr EA, Timpson NJ, Andersen CS, Davey Smith G, Olsen J, Sorensen TI. Severe obesity in young women and reproductive health: the Danish National Birth Cohort. PLoS One 2009;4:e8444.

11. Paternoster L, Evans DM, Nohr EA, et al. Genome-wide population-based association study of extremely overweight young adults--the GOYA study. PLoS One 2011;6:e24303.

12. Zhang G, Bacelis J, Lengyel C, et al. Assessing the Causal Relationship of Maternal Height on Birth Size and Gestational Age at Birth: A Mendelian Randomization Analysis. PLoS medicine 2015;12:e1001865.

13. Carvalho B, Bengtsson H, Speed TP, Irizarry RA. Exploration, normalization, and genotype calls of high-density oligonucleotide SNP array data. Biostatistics 2007;8:485-99.

14. Scharpf RB, Irizarry RA, Ritchie ME, Carvalho B, Ruczinski I. Using the R Package crlmm for Genotyping and Copy Number Estimation. Journal of statistical software 2011;40:1-32.

15. Delaneau O, Marchini J, Zagury JF. A linear complexity phasing method for thousands of genomes. Nature methods 2012;9:179-81.

16. Genomes Project C, Abecasis GR, Auton A, et al. An integrated map of genetic variation from 1,092 human genomes. Nature 2012;491:56-65.

17. Marchini J, Howie B. Genotype imputation for genome-wide association studies. Nature reviews Genetics 2010;11:499-511.

18. Hendricks AE, Dupuis J, Logue MW, Myers RH, Lunetta KL. Correction for multiple testing in a gene region. Eur J Hum Genet 2014;22:414-8.

19. Gao X, Starmer J, Martin ER. A multiple testing correction method for genetic association studies using correlated single nucleotide polymorphisms. Genetic epidemiology 2008;32:361-9.

20. Welter D, MacArthur J, Morales J, et al. The NHGRI GWAS Catalog, a curated resource of SNP-trait associations. Nucleic Acids Res 2014;42:D1001-6.

21. Consortium GT. The Genotype-Tissue Expression (GTEx) project. Nature genetics 2013;45:580-5.

22. Yang J, Ferreira T, Morris AP, et al. Conditional and joint multiple-SNP analysis of GWAS summary statistics identifies additional variants influencing complex traits. Nature genetics 2012;44:369-75, S1-3.

23. Zhang G, Karns R, Sun G, et al. Finding missing heritability in less significant Loci and allelic heterogeneity: genetic variation in human height. PLoS One 2012;7:e51211.

24. Yang J, Lee SH, Goddard ME, Visscher PM. GCTA: a tool for genome-wide complex trait analysis. American journal of human genetics 2011;88:76-82.

25. de Leeuw CA, Mooij JM, Heskes T, Posthuma D. MAGMA: generalized gene-set analysis of GWAS data. PLoS Comput Biol 2015;11:e1004219.

26. Wagner GP, Kin K, Lynch VJ. Measurement of mRNA abundance using RNA-seq data: RPKM measure is inconsistent among samples. Theory Biosci 2012;131:281-5.

27. Weirauch MT, Yang A, Albu M, et al. Determination and inference of eukaryotic transcription factor sequence specificity. Cell 2014;158:1431-43.

28. Johnson DS, Mortazavi A, Myers RM, Wold B. Genome-wide mapping of in vivo protein-DNA interactions. Science 2007;316:1497-502.

29. Lynch VJ, Nnamani MC, Kapusta A, et al. Ancient transposable elements transformed the uterine regulatory landscape and transcriptome during the evolution of mammalian pregnancy. Cell Rep 2015;10:551-61.

30. Langmead B, Salzberg SL. Fast gapped-read alignment with Bowtie 2. Nature methods 2012;9:357-9.

31. Langmead B, Trapnell C, Pop M, Salzberg SL. Ultrafast and memory-efficient alignment of short DNA sequences to the human genome. Genome Biol 2009;10:R25.

32. Brohee S, Bontempi G. D-peaks: a visual tool to display ChIP-seq peaks along the genome. Transcription 2012;3:255-9.

33. Buenrostro JD, Giresi PG, Zaba LC, Chang HY, Greenleaf WJ. Transposition of native chromatin for fast and sensitive epigenomic profiling of open chromatin, DNA-binding proteins and nucleosome position. Nature methods 2013;10:1213-8.

34. Miller DE, Patel ZH, Lu X, Lynch AT, Weirauch MT, Kottyan LC. Screening for Functional Non-coding Genetic Variants Using Electrophoretic Mobility Shift Assay (EMSA) and DNA-affinity Precipitation Assay (DAPA). J Vis Exp 2016.
